# Supplementary material for: Direct synthesis of cyanate anion from dinitrogen catalysed by molybdenum complexes bearing pincer-type ligand
Source: Nat Commun. 2022 Oct 24;13:6161. doi: 10.1038/s41467-022-33809-5 (PMC9592615; doi:10.1038/s41467-022-33809-5)
Supplement: Supplementary file 1 — Supplementary Information [file 41467_2022_33809_MOESM1_ESM.pdf]

Supplementary Information

Direct synthesis of cyanate anion from dinitrogen catalysed by molybdenum complexes bearing pincer-type ligand

Takayuki Itabashi,[1] Kazuya Arashiba,[1] Akihito Egi,[2] Hiromasa Tanaka,[3] Keita Sugiyama,[1] Shun Suginome,[1] Shogo Kuriyama,[1] Kazunari Yoshizawa,*[2] Yoshiaki Nishibayashi*[1]

[1]Department of Applied Chemistry, School of Engineering, The University of Tokyo, Hongo, Bunkyo-ku, Tokyo 113-8656, Japan

[2]Institute for Materials Chemistry and Engineering, Kyushu University, Nishi-ku, Fukuoka 819-0395, Japan

[3]School of Liberal Arts and Sciences, Daido University, Takiharu-cho, Minami-ku, Nagoya 457-8530, Japan

Contents

Supplementary Methods p. S2
- **General Methods** p. S2
- **Experimental Section** p. S2
- **Electrochemistry** p. S6
- **X-ray Crystallographic Studies** p. S6
- **Computational Details** p. S8

Supplementary Figures p. S10
- **Cyclic Voltammograms** p. S13
- **NMR Spectra** p. S14
- **IR Spectra** p. S17
- **X-ray Crystal Structures** p. S20
- **Optimized Structures** p. S27

Supplementary Tables p. S33

Supplementary References p. S38

Supplementary Methods

General Methods.

^{1}H NMR (400 MHz), ^{31}P{^{1}H} NMR (162 MHz) and ^{15}N{^{1}H} NMR (41 MHz) spectra were recorded on a JEOL ECS-400 spectrometer in suitable solvent, and spectra were referenced to residual solvent (^{1}H) or external standard (^{31}P{^{1}H}: 85% H_3PO_4, ^{15}N{^{1}H}: CH_3NO_2). Magnetic susceptibility was measured using the Evans method.[1] IR spectra were recorded on a JASCO FT/IR 4100 Fourier transform infrared spectrometer. ATR-IR spectra were recorded on a SHIMADZU IR Spirit equipped with a QATR-S ATR unit. Mass spectra were recorded on a JEOL Accu TOF JMS-T100LP. Ion chromatography analyses were performed on TOSOH IC8100 equipped with TSKgel SuperIC-Anion HS column and TSKgel guardcolumn SuperIC-A HS. Cyclic voltammograms were recorded on an ALS/Chi model 610C electrochemical analyzer. Elemental analyses were performed at Microanalytical Center of The University of Tokyo.

All manipulations were carried out under an atmosphere of nitrogen or argon by using standard Schlenk techniques or glovebox techniques unless otherwise stated. Solvents were dried by general methods, and degassed before use. [MoI_3(PNP)] (PNP = 2,6-bis(di-*tert*-butylphosphinomethyl)pyridine),[2] PhOCOI,[3] $CoCp^*_2$,[4] KC_8,[5] $SmI_2(thf)_2$,[6] $SmI_3(thf)_{3.5}$,[7] AgNCO,[8] potassium 3,5-bis(trifluoromethyl)phenoxide,[9] [Mo(N)Cl(PPP)] (**5**, PPP = bis(di-*tert*-butylphosphinoethyl)phenylphosphine),[10] and [Mo(N)I(PCP)] (**6**, PCP = 1,3-bis(di-*tert*-butylphosphinomethyl)benzimidazole-2-ylidene)[11] were prepared according to the literature methods. Other reagents were purchased commercially and used as received.

Experimental Section.

Synthesis of [Mo(N)I(PNP)] (1). [$MoI_3(PNP)_3$] was prepared according to the literature method as follows.[2] A mixture of [$MoI_3(thf)_3$] and PNP in THF was stirred at 50 °C for 18 h. The resultant reddish brown solution was filtered through Celite, and the filter cake was washed with THF. The combined filtrate was concentrated in vacuo, and the residue was washed with Et_2O to afford [$MoI_3(PNP)_3$] as a brown solid. KC_8 was prepared by stirring a mixture of graphite and potassium at 200 °C for 20 min.[5] To a mixture of [MoI_3(PNP)] (347 mg, 0.398 mmol) and KC_8 (120.0 mg, 0.888 mmol) was added cooled toluene (20 mL, −78 °C) under N_2 (1 atm). After stirring at −78 °C for 5 min, the mixture was warmed to room temperature and further stirred at room temperature for 18 h. Volatiles were removed *in vacuo*, and the residue was washed with Et_2O (5 mL x 3). Then, THF (15 mL) was added to the residue. The solution was filtered through Celite, and the filter cake was washed with THF (5 mL x 3). After the combined filtrate was concentrated to about 20 mL, slow addition of hexane (50 ml) afforded **1** as brown crystals, which were collected by filtration, washed with Et_2O (4 mL x 3), and dried *in vacuo* (96.8 mg, 0.153 mmol, 38% yield). Spectroscopic data were identified with previously reported literature.[2]

Synthesis of [Mo(^{15}N)I(PNP)] (1-^{15}N). Complex **1-^{15}N** was synthesized in a manner similar to that of **1**. The reaction of [MoI_3(PNP)] (347 mg, 0.398 mmol) with KC_8 (130.0 mg, 0.961 mmol) under $^{15}N_2$ gave **1-^{15}N** (79.0 mg, 0.125 mmol, 31% yield). Spectroscopic data were identified with previously reported literature.[2]

Synthesis of [Mo(NCOOPh)ClI(PNP)] (2). A mixture of **1** (96.1 mg, 0.152 mmol) and phenyl chloroformate

(26.2 mg, 0.167 mmol) in THF (15 mL) was stirred at room temperature for 2 h. The resultant brown solution was filtered through Celite, and the filter cake was washed with THF (1 mL x 3). After the filtrates were combined, slow addition of hexane (35 mL) afforded **2**·THF as brown crystals, which were collected by filtration, washed with ether (4 mL x 3), and dried *in vacuo* (108.5 mg, 0.126 mmol, 83% yield). ^{1}H NMR (CD_3CN): δ 7.95 (t, J = 7.8 Hz, OPh, 1H), 7.77 (d, J = 7.8 Hz, OPh, 2H), 7.40 (t, J = 7.8 Hz, Ar*H*, 2H), 7.29 (t, J = 7.1 Hz, Ar*H*, 1H), 6.98 (d, J = 7.8 Hz, OPh, 2H), 4.19 (dt, J = 16.5, 4.4 Hz, CH_2P, 2H), 3.88 (dt, J = 16.5, 3.4 Hz, CH_2P, 2H), 1.44 (t, J = 6.6 Hz, $P^{t}Bu_2$, 18H), 1.36 (t, J = 6.6 Hz, $P^{t}Bu_2$, 18H). ^{31}P{^{1}H} NMR (CD_3CN): δ 73.6 (s). IR(ATR-IR): 1690 cm^{-1} ($\nu_{C=O}$) and 642 cm^{-1} ($\nu_{Mo=N}$). Anal. Calcd. for $C_{30}H_{48}ClIMoN_2O_2P_2\cdot C_4H_8O$: C, 47.43; H, 6.56; N, 3.25. Found: C, 47.05; H, 6.45; N, 3.39.

Synthesis of [Mo(^{15}NCOOPh)ClI(PNP)] (2-^{15}N). Complex **2-^{15}N** was synthesized in a manner similar to that of **2** using **1-^{15}N** (72.2 mg, 0.114 mmol) to give [Mo(^{15}NCOOPh)(Cl)(I)(PNP)] (**2-^{15}N**)·THF (81.2 mg, 0.0942 mmol, 83% yield). ^{15}N{^{1}H} NMR (CD_3CN): δ -51.6 (t, $J_{N\text{-}P}$ = 4.9 Hz). IR(ATR-IR): 1690 cm^{-1} ($\nu_{C=O}$) and 633 cm^{-1} ($\nu_{Mo=15N}$).

Reaction of 1 with PhOCOI. PhOCOI was prepared by the reaction of PhOCOCl with NaI in acetonitrile at 70 °C.[3] A mixture of **1** (12.6 mg, 0.020 mmol) and PhOCOI (5.5 mg, 0.022 mmol) in THF (1.5 mL) was stirred at room temperature for 30 min. Volatiles were removed *in vacuo*, the residue was dissolved in CH_2Cl_2 (1 mL). Slow addition of hexane (3 mL) afforded [Mo(N)I(PCP)][I] ([**1**][I]) as red crystals, which were collected by filtration, washed with ether (4 mL x 3), and dried *in vacuo* (15.0 mg, 0.020 mmol, 99% yield). Magnetic susceptibility (Evans method): μ_{eff} = 1.9 μ_B in CD_2Cl_2 at 294 K. Anal. Calcd. for $C_{23}H_{43}I_2MoN_2P_2$: C, 36.38; H, 5.71; N, 3.69. Found: C, 36.73; H, 5.31; N, 3.36.

Reduction of 2 with SmI_2 under N_2. $SmI_2(thf)_2$ was prepared by stirring a mixture of Sm metal and diiodoethane in THF at 60 °C overnight.[6] A mixture of **2**·THF (8.7 mg, 0.010 mmol) and $SmI_2(thf)_2$ (28.1 mg, 0.051 mmol) in THF (10 mL) was stirred at room temperature for 2 h under N_2 (1 atm). After volatiles were removed *in vacuo*, the yield of **1** and [SmI_2(OPh)] (**3a**) in the residual solid was determined by ^{1}H NMR in THF-d_8, respectively, using hexamethylbenzene as an internal standard (74% NMR yield for **1**, 93% NMR yield for **3a**). ESI-TOF-MS of the residual solid in THF showed ion peaks at m/z = 634.17, which were assigned as those of **1** (Supplementary Figure 1). $KO^{t}Bu$ (0.25 mmol) in THF (2 mL) was added to the resultant mixture, and the mixture was stirred at room temperature for 20 min. The volatiles were removed *in vacuo*. The IR spectrum of the residual solid was measured (IR (KBr) 2170 cm^{-1}, ν_{NCO}, Supplementary Figure 2). A KOH aqueous solution (0.1 M, 25 mL) was added to the residual solid, and the mixture was stirred at room temperature for 30 min. The supernatant of white suspension was filtered through OnGuard II Na, the yield of NCO^{-} in residual solution was determined by ion chromatography (75% IC yield).

Reduction of 2-^{15}N with SmI_2 under N_2. The reduction of **2-^{15}N** with $SmI_2(thf)_2$ was performed in a manner similar to that of **2** described above under $^{14}N_2$ atmosphere (1 atm) to give [Mo(^{14}N)I(PNP)] (**1**) in 41% NMR yield

(ESI-TOF-MS: found m/z = 634.18, Supplementary Figure 3), **3a** in 97% NMR yield, and $^{15}NCO^{-}$ in 62% IC yield (IR (KBr) 2153 cm^{-1}, ν_{15NCO}, Supplementary Figure 4).

Synthesis of [SmI$_2$(OPh)] (3a). SmI$_3$(thf)$_{3.5}$ was prepared by stirring a mixture of Sm metal and iodine in THF at room temperature for three days.[7] A mixture of SmI$_3$(thf)$_{3.5}$ (15.2 mg, 0.0194 mmol) and KOPh (2.6 mg, 0.0196 mmol) in THF was stirred at room temperature for 18 h. The resultant yellow suspension was filtered through Celite, and the filter cake was washed with THF (2 mL x 3). After the filtrate was concentrated to 2 mL, slow addition of hexane (6 mL) afforded [SmI$_2$(OPh)(thf)$_4$] as colorless crystals, which were collected by filtration, washed with pentane (1 mL x 3). Obtained crystals were dried *in vacuo* to afford pale yellow solid (6.4 mg, 0.010 mmol, 51% yield). Elemental analysis of the pale yellow solid indicated dissociation of two molecules of thf. ^{1}H NMR (THF-d_8): δ 8.96 (d, J = 6.4 Hz, OPh, 2H), 7.60 (t, J = 6.4 Hz, OPh, 2H), 7.22 (t, J = 6.4 Hz, OPh, 1H). Anal. Calcd. for $C_{14}H_{21}I_2O_3Sm$ ([SmI$_2$(OPh)(thf)$_2$]): C, 26.21; H, 3.30. Found: C, 26.59; H, 3.69.

Synthesis of {[SmI(NCO)(18-crown-6)][I]}$_n$ (3b). AgNCO was prepared by the reaction of sodium cyanate with silver nitrate in water at room temperature overnight.[8] A mixture of SmI$_2$(thf)$_2$ (54.8 mg, 0.102 mmol) and 18-crown-6 (26.9 mg, 0.102 mmol) in THF (10 mL) was stirred at room temperature for 1 h. After the reaction, volatiles were removed *in vacuo*. The residue was dissolved in MeCN (6 mL), and then AgNCO (16.1 mg, 0.107 mmol) was added to the green solution and the reaction mixture was stirred at room temperature for 20 min. After the reaction, the reaction mixture was filtered through Celite, and the filter cake was washed with MeCN (1 mL x 2). After the filtrates were combined, slow addition of Et$_2$O (15 mL) afforded·**3b**·(MeCN)$_n$ as colorless crystals, which were collected by filtration, washed with Et$_2$O (3 mL x 3). Obtained crystals were dried *in vacuo* to afford pale yellow solid (46.2 mg, 0.0615 mmol, 60% yield). Hydrolysis of **3b** with a KOH aqueous solution (0.1 M, 25 mL) at room temperature for 30 min gave NCO^{-} quantitatively (>99% IC yield). ^{1}H NMR (CD$_3$CN): δ 4.37 (br s, CH_2, 2H), 3.66 (br s, CH_2, 8H), 3.50 (br s, CH_2, 8H), 2.88 (br s, CH_2, 6H). IR(ATR-IR): 2232, 2182, 2165 cm^{-1} (ν_{NCO}, ν_{CN}). Anal. Calcd. for $C_{15}H_{27}I_2N_2O_7Sm$: C, 23.97; H, 3.62; N, 3.73. Found: C, 24.14; H, 3.44; N, 3.82.

Synthesis of [Mo(NCO)ICl(PNP)] (4a). Potassium 3,5-bis(trifluoromethyl)phenoxide was prepared by the reaction of 3,5-bis(trifluoromethyl)phenol with potassium hydride in THF at 70 °C overnight.[9] A mixture of **2**·THF (77.7 mg, 0.0902 mmol) and SmI$_2$(thf)$_2$ (50.1 mg, 0.0930 mmol) in THF (8 mL) was stirred at room temperature for 10 min under an atmospheric pressure of argon. After the reaction, potassium 3,5-bis(trifluoromethyl)phenoxide (55.6 mg, 0.207 mmol) was added to the orange solution, and the mixture was stirred at room temperature for 10 min. Volatiles were removed *in vacuo*, and the residue was dissolved in THF (3 mL). The solution was filtered through Celite, and the filter cake was washed with THF (1 mL x 2). After the filtrates were combined, slow addition of Et$_2$O (15 mL) afforded **4a**·0.5THF as orange crystals, which were collected by filtration, washed with ether (5 mL x 3), and dried *in vacuo* (45.1 mg, 0.0615 mmol, 68% yield). Magnetic susceptibility (Evans method): μ_{eff} = 3.7 μ_B in THF-d_8 at 294 K. IR(ATR-IR): 2209 cm^{-1} (ν_{NCO}). Anal. Calcd. for $C_{26}H_{47}ClIMoN_2O_{1.5}P_2$: C, 42.67; H, 6.47; N, 3.83. Found: C, 42.73; H, 6.48; N, 3.62.

Synthesis of [Mo(^{15}NCO)ICl(PNP)] (4a-^{15}N). Complex **4a-^{15}N** was synthesized in a manner similar to that of **4a** using **2-^{15}N**·THF (43.0 mg, 0.0499 mmol) to give **4a-^{15}N**·0.5THF (17.1 mg, 0.0233 mmol, 47% yield). IR(ATR-IR): 2195 cm^{-1} (ν_{15NCO}).

Synthesis of [Mo(NCO)(OPh)Cl(PNP)] (4b). CoCp*$_2$ was prepared from [CoCp*$_2$][PF_6] and a slight excess of KC_8 in THF over 4 h.[4] A mixture of **2**·THF (32.4 mg, 0.038 mmol) and CoCp*$_2$ (14.1 mg, 0.043 mmol) in THF (12 mL) was stirred at room temperature for 16 h under an atmospheric pressure of argon. Volatiles were removed *in vacuo*, and THF (3 mL) was added to the residue. The solution was filtered through Celite, and the filter cake was washed with THF (1 mL x 2). After the filtrates were combined, slow addition of hexane (10 mL) afforded **4b** as orange crystals, which were collected by filtration, washed with ether (2 mL x 3), and dried *in vacuo* (12.1 mg, 0.018 mmol, 49% yield). Magnetic susceptibility (Evans method): μ_{eff} = 3.9 μ_{B} in THF-d_8 at 294 K. IR(ATR-IR): 2214 cm^{-1} (ν_{NCO}). Anal. Calcd. for $C_{30}H_{48}ClMoN_2O_2P_2$: C, 54.42; H, 7.31; N, 4.23. Found: C, 54.06; H, 7.52; N, 4.43.

Reduction of 4a with SmI_2 under N_2. The reduction of **4a** with $SmI_2(thf)_2$ was performed in a manner similar to that of **2** described above under N_2 atmosphere (1 atm) to give **1** in 66% NMR yield and NCO^- in 75% IC yield.

Reduction of 4b with CoCp*$_2$ under N_2 atmosphere. A mixture of **4b** (6.7 mg, 0.010 mmol), CoCp*$_2$ (10.1 mg, 0.031 mmol) and NaI (4.5 mg, 0.030 mmol) in THF (2 mL) was stirred at room temperature for 16 h. The resultant mixture was filtered through Celite, and the filtrate was concentrated *in vacuo*. ESI-TOF-MS of the residue in THF showed some ion peaks at m/z = 1166.6, 1224.7, 1282.8, which were assigned as that of [Mo(NCO)(X)(PNP)](μ-N_2)[Mo(NCO)(X')(PNP)] (X, X' = Cl; m/z = 1166.3 , X = Cl and X' = OPh; m/z = 1224.4, X, X' = OPh; m/z = 1282.5), respectively. Recrystallization of the residue from THF-hexane afforded [Mo(NCO)Cl(PNP)]$_2$(μ-N_2) as brown crystals.

Synthesis of [Mo(N)I(PPP)] (5).[10] A mixture of [$MoCl_3$(thf)$_3$] and Me_3SiN_3 in THF was stirred at 50 °C for 1 h. The resultant solution was concentrated under reduced pressure. To the residue was added to a solution of PPP in THF, and then the mixture was stirred at 50 °C for 18 h. After cooling at room temperature, to the solution was added KC_8 and stirred for 23 h at room temperature. The resultant solution was filtered through Celite, and the filter cake was washed with THF. The combined filtrate was concentrated in vacuo, and the residue was washed with Et_2O to afford **5**.

Synthesis of [Mo(N)I(PCP)] (6).[11] To a mixture of [MoI_3(PCP)] and CoCp*$_2$ was added cooled THF (−78 °C) under N_2 (1 atm). After stirring at −78 °C for 5 min, the mixture was warmed to room temperature and further stirred at room temperature for 18 h. Volatiles were removed *in vacuo*, and the residue was washed with Et_2O. Then, THF was added to the residue. The solution was filtered through Celite, and the filter cake was washed with THF. After the combined filtrate was concentrated, slow addition of hexane afforded **1**.

Trials of Catalytic Reaction.

One-Portion Addition. A typical experimental procedure is described below. To a mixture of **1** (6.3 mg, 0.010 mmol) and $SmI_2(thf)_2$ (198 mg, 0.36 mmol) was added THF (3 mL) under N_2. Then, a THF solution (2 mL) containing phenyl chloroformate (18.8 mg, 0.12 mmol) was added, and the mixture was stirred at room temperature for 16 h. After the reaction, volatiles were removed *in vacuo*. A KOH aqueous solution (0.1 M, 25 mL) was added to the residual solid, and the mixture was stirred at room temperature for 30 min. The supernatant of the suspension was filtered through OnGuard II Na, the yields of NCO^- in residual solution was determined by ion chromatography.

Slow Addition of Chloroformate Ester via Syringe Pump. A typical experimental procedure is described below. In a nitrogen-filled glove box, to a mixture of **6** (6.7 mg, 0.010 mmol) and $SmI_2(thf)_2$ (198 mg, 0.36 mmol) in a 50 mL Schlenk flask was added THF (5 mL) under N_2. Then a solution of phenyl chloroformate (18.8 mg, 0.12 mmol) in THF (3 mL) was slowly added to the stirred solution in the Schlenk flask with a syringe pump over a period of 3 h (slow addition time). After the addition of phenyl chloroformate, the mixture was further stirred at room temperature for additional 13 h (total 16 h). After the reaction, volatiles were removed *in vacuo*. A KOH aqueous solution (0.1 M, 25 mL) was added to the residual solid, and the mixture was stirred at room temperature for 30 min. The supernatant of the suspension was filtered through OnGuard II Na, the yields of NCO^- in residual solution was determined by ion chromatography.

Electrochemistry.

Cyclic voltammograms were recorded on an ALS/Chi model 610C electrochemical analyzer with platinum working electrode in THF containing 1 mM of sample and 0.1 M of $[^{n}Bu_4N][PF_6]$ as a supporting electrolyte at a scan rate of 0.1 V/s at room temperature under Ar atmosphere. All potentials were measured against an $Ag^{0/+}$ electrode and converted to the values *vs*. ferrocene/ferrocenium ($FeCp_2^{0/+}$) referenced to external standard ($FeCp_2^{0/+}$). Cyclic voltammograms of **2** and **4a** are shown in Supplementary Figures 6 and 7, respectively.

X-Ray Crystallographic Studies.

Diffraction data for **[1]I**, **2**, **3a**, **3b**·$n$$CH_3CN$, **4a**, **4b**, and $[Mo(NCO)Cl(PNP)]_2(\mu\text{-}N_2)$ were collected for the 2θ range of 4° to 60° at – 180 °C on a Rigaku XtaLAB Synergy-S diffractometer equipped with a HyPix-6000HE Hybrid Photon Counting (HPC) detector and VariMax optics using multi-layer mirror monochromated Mo Kα radiation (λ = 0.71073 Å). Intensity data were corrected for Lorenz-polarization effects and for empirical absorption (CrysAlisPro),[12] whereas structure solutions and refinements were carried out by using the *CrystalStructure* crystallographic software package.[13] Positions of non-hydrogen atoms were determined by direct methods (SHELXT Version 2014/5 for **[1]I**, **3a**, **3b**·$n$$CH_3CN$, and **4b**, SHELXS Version 2013/1 for **2**, **4a**, and $[Mo(NCO)Cl(PNP)]_2(\mu\text{-}N_2)$)[14,15] and subsequent Fourier syntheses (SHELXL Version 2016/6),[16] and were refined on F_o^2 using all unique reflections by full-matrix least-squares with anisotropic thermal parameters except for several disordered atoms summarized below, which were solved isotropically. All the other hydrogen atoms were placed at the calculated positions with fixed isotropic parameter except for several hydrogen atoms summarized below, which could not be placed at calculated positions mainly because of disorders. Anomalous dispersion effects were

included in F_c,[17] and mass attenuation coefficients, values for $\Delta f'$ and $\Delta f''$, and neutral atom scattering factors were taken from references.[18–20] Details of the crystal and data collection parameters are summarized in Supplementary Tables 2–4, and ORTEP drawings of molecular structures are shown in Supplementary Figures 19–25.

Three methyl carbon atoms in **[1]I** were solved as disorders (C17A and C17B, C18A and C18B, C19A and C19B, solved in a ratio of 0.5:0.5, respectively) with isotropic thermal ellipsoids and with nine hydrogen atoms unable to be located at calculated positions because of such disorders. One molybdenum atom in **[1]I** was solved as a disorder between two places (Mo1A and Mo1B, solved in a ratio of 0.95:0.05), which were refined anisotropically. One disordered methyl carbon atom (C21) was solved anisotropically with neglecting its minor disorder, although three hydrogen atoms attached to the C21 atom could not be located at calculated positions, because the solved position of C21 was too near to the position of minor disordered molybdenum atom (Mo1B), leading to a B-level alert (PLAT315_ALERT_2_B Singly Bonded Carbon Detected (H-atoms Missing), C21). In total, twelve hydrogen atoms could not be located at calculated positions in **[1]I**, leading to another B-level alert (PLAT043_ALERT_1_B Calculated and Reported Mol. Weight Differ by 12.12).

3a was solved as a mixture of [SmI_2(OPh)(thf)$_4$] (I1A, I2A, I4A, I5A, O1, O4, C1, C2, C3, C4, C5, C15, C16, C17, C18, C19, C20) and [SmI_3(thf)$_4$] (I1B, I2B, I3, I4B, I5B, I6) (both consisting of two crystallographically independent molecules) in a ratio of 0.92:0.08, where I3, C1, C2, C3, C4, C5 atoms were refined isotropically with ten hydrogen atoms attached to OPh moieties (9.2 hydrogen atoms per asymmetric unit) unable to be located at calculated positions because of such disorders. Two carbon atoms of two of the thf ligands for each crystallographically independent molecule in **3a** were also disordered between two places (C7A and C7B, C10A and C10B, C21A and C22B, C24A and C24B, solved in a ratio of 0.5:0.5, respectively), thus thirty-two hydrogen atoms could not be located at calculated positions because of such disorders. In total, 41.2 hydrogen atoms per asymmetric unit, or 20.6 hydrogen atoms per Sm could not be located in **3a**, leading to a B-level alert (PLAT043_ALERT_1_B Calculated and Reported Mol. Weight Differ by 20.82).

There are several B-level alerts for crystallographic data of **3b**·$n$$CH_3CN$ (PLAT971_ALERT_2_B Check Calcd Resid. Dens. 1.04Ang From I1, 2.70 eÅ^{-3}, PLAT972_ALERT_2_B Check Calcd Resid. Dens. 0.39Ang From I1, -3.48 eÅ^{-3}, PLAT972_ALERT_2_B Check Calcd Resid. Dens. 0.33Ang From I1, -3.37 eÅ^{-3}, PLAT973_ALERT_2_B Check Calcd Positive Resid. Density on Sm1, 1.61 eÅ^{-3}). These are due to several minor disorders of Sm1 and I1 atoms, although such minor disorders were not solved here.

Positions of nitrogen and oxygen atoms of cyanate moieties in **3b** were determined based on the difference Fourier electron density map, where nitrogen and oxygen atoms solved at the position in the current solution gave lower R_1 and wR_2 values compared to those in inverse.

In **4a**, it is difficult to determine the positions of nitrogen and oxygen atoms based on the difference Fourier electron density map because of the disorder between chloride and cyanate ligands. However, from the reaction of nitride complex with phenyl chloroformate (Figure 3a), it is considered that the nitrogen atom of the cyanate ligand binds to the Mo atom. In addition, DFT calculations were carried out to optimize the structures for both binding modes (Supplementary Figure 29) and to conclude that the nitrogen atom in cyanate ligand binds to Mo atom.

Chlorine and cyanate moieties in one of the two crystallographically independent molecules in **4a** were solved as disorders between two places (Cl2A and Cl2B, O2A and O2B, solved in a ratio of 0.9:0.1, whereas minor disorders

of N4A and C48A (each solved with 0.9 atom occupancy) were not solved) with Cl2B and O2B atoms refined isotropically.

One of two pairs of chlorine and cyanate moieties in $[Mo(NCO)Cl(PNP)]_2(\mu\text{-}N_2)$ was solved as a disorder between two places (Cl2A and Cl2B, O2A and O2B, N6A and N6B, and C48A and C48B, solved in a ratio of 0.5:0.5), all refined anisotropically.

The unit cells of **2**, **4a,** and $[Mo(NCO)Cl(PNP)]_2(\mu\text{-}N_2)$ contain solvent accessible voids of 426, 1904, and 531 $Å^3$, respectively. The difference Fourier maps suggested that voids of **2** and **4a** were occupied by a THF molecule per asymmetric unit, which could not be located appropriately because of heavy disorders. The difference Fourier maps suggested that voids of $[Mo(NCO)Cl(PNP)]_2(\mu\text{-}N_2)$ were occupied by one and a half of hexane molecules per asymmetric unit, which could not be located appropriately because of heavy disorders. The electron density associated with these solvent molecules was removed by the SQUEEZE routine of PLATON[S21] for crystal data of **2**, **4a**, and $[Mo(NCO)Cl(PNP)]_2(\mu\text{-}N_2)$.

Computational Details.

DFT calculations were performed with the Gaussian16 program (Rev. C.01).[22] Geometry optimizations were carried out with the B3LYP-D3 functional, which is the B3LYP hybrid functional[23–26] combined with an empirical dispersion correction developed by Grimme.[27] In the present calculations, the SDD (Stuttgart/Dresden pseudopotentials) basis sets[28,29] are employed for the molybdenum, iodine, and chlorine atoms. The 6-31G(d) basis sets[30–33] are employed for the other atoms. All stationary-point structures were confirmed to have the appropriate number of imaginary frequencies by vibrational analysis. An appropriate connection between a reactant and a product was confirmed by IRC[34–36] and quasi-IRC calculations. In the quasi-IRC calculation, the geometry of a transition state was at first shifted by perturbing the geometries very slightly along the reaction coordinate and released for equilibrium optimization. To determine free energy profiles, single-point energy calculations were performed at the optimized geometries using the SDD and 6-311+G(d,p) basis sets.[37–39] In the single-point calculations, solvation effects of THF (ε = 7.4257) were taken into account by using the polarizable continuum model (PCM).[40]

Supplementary Figure 26 depicts optimized structures of **RC**, $\mathbf{TS_{1/2}}$, **PC**, and **2** in Figure 5a. Supplementary Figure 27 shows free energy profiles at 298 K (ΔG_{298}) of the carbon-oxygen bond cleavage of **I** to afford isocyanate complex **4a** in the doublet and quartet states. In the quartet spin state, this reaction has virtually no activation barrier because the consideration of solvent effects in the single-point calculations resulted in a negative value of the activation free energy ($\Delta G_{298}^{\ddagger}$ = –2.6 kcal/mol). Energy differences *in vacuo* without thermal corrections are described in Supplementary Figure 27. Supplementary Figure 28 shows optimized structures of **4a**, MoICl(PNP), and intermediates described in Figure 6 in the ground state. Supplementary Figure 29 presents an optimized structure of **4a′** with the Mo-OCN binding mode, a linkage isomer of **4a** with the Mo-NCO binding mode. The isocyanate complex **4a** is 14.4 kcal/mol more stable than the cyanate complex **4a′** at 298 K. In addition, **4a** has a linear Mo-NCO moiety (Mo-N-C = 175.9°), while **4a′** has a bent Mo-OCN moiety (Mo-O-C = 131.4°). Supplementary Figure 30 shows optimized structures of samarium(III) complexes, $[SmI_2(THF)_5]^+$, $SmI_2(NCO)(THF)_4$, and $SmI_2(OPh)(THF)_4$. The structures of the Sm(III) species in THF were based on the X-ray crystallographic structure of $[Sm^{II}I_2(THF)_5]$.[41]

Detailed data on SCF energies, thermal energy corrections at 298 K, and SCF energies in THF are summarized in Supplementary Table 5. Cartesian coordinates of all optimized structures are given in a separated .xyz file.

Supplementary Figures

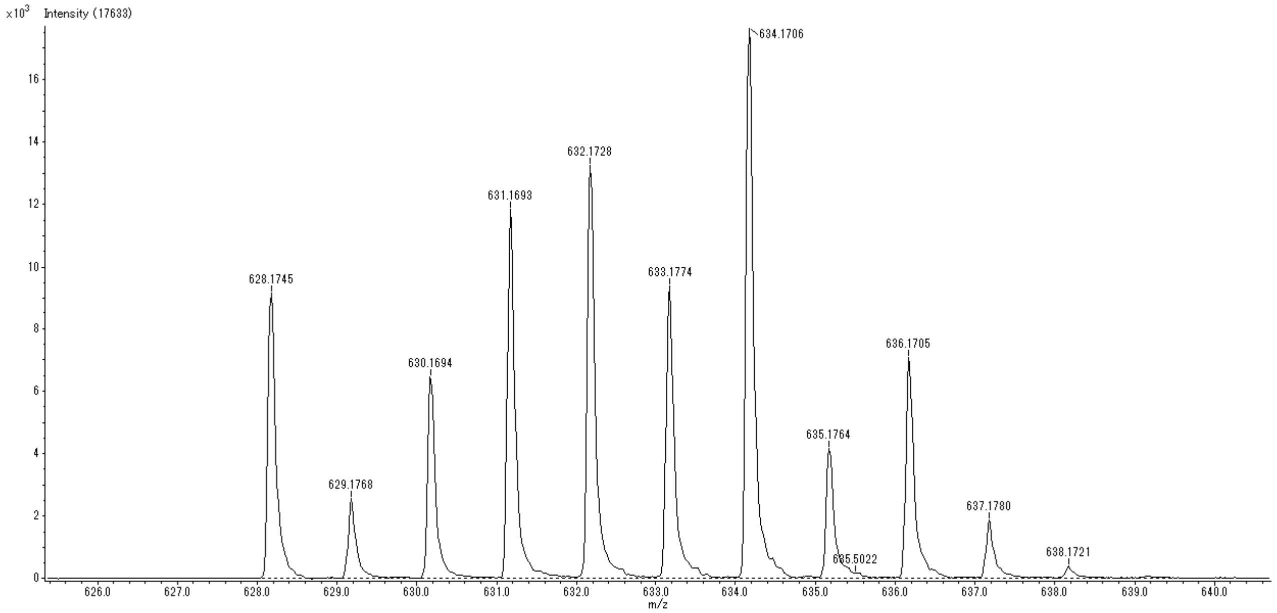

Supplementary Figure 1. ESI-TOF-MS spectrum of the residue obtained from the reduction of **2** with SmI_2 under N_2.

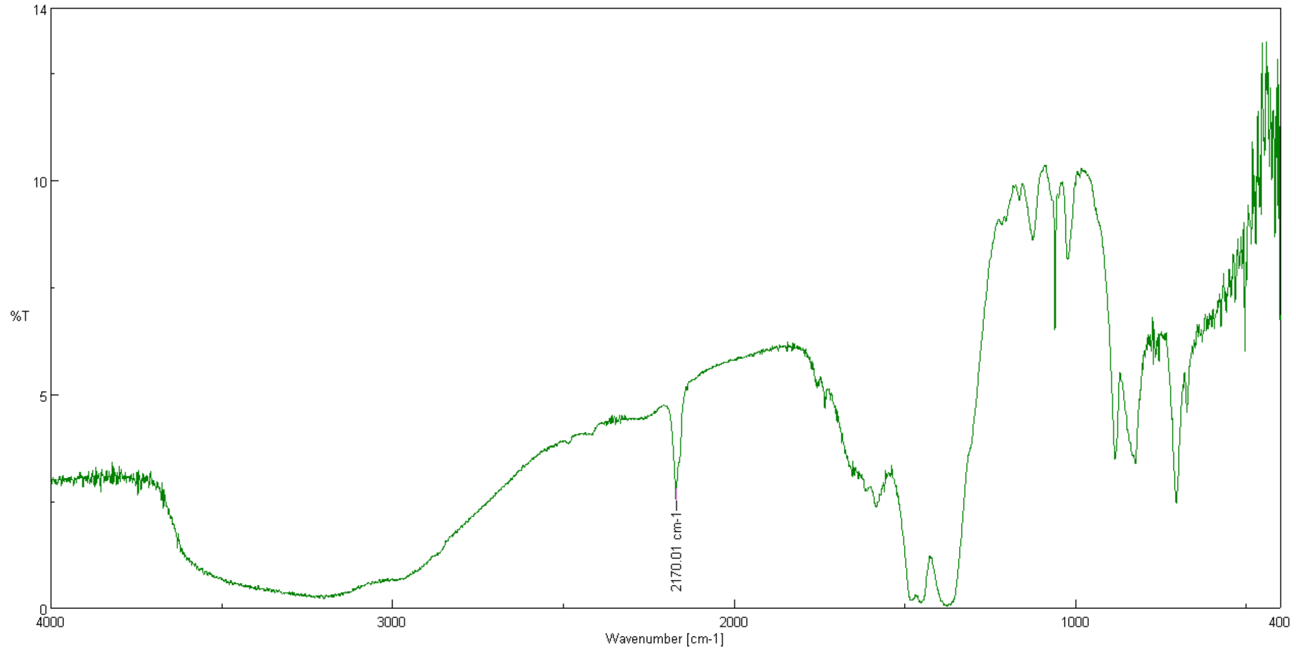

Supplementary Figure 2. IR spectrum of the residual solid obtained after treating the reaction of **2** with SmI_2 under N_2 with KO^tBu (in KBr).

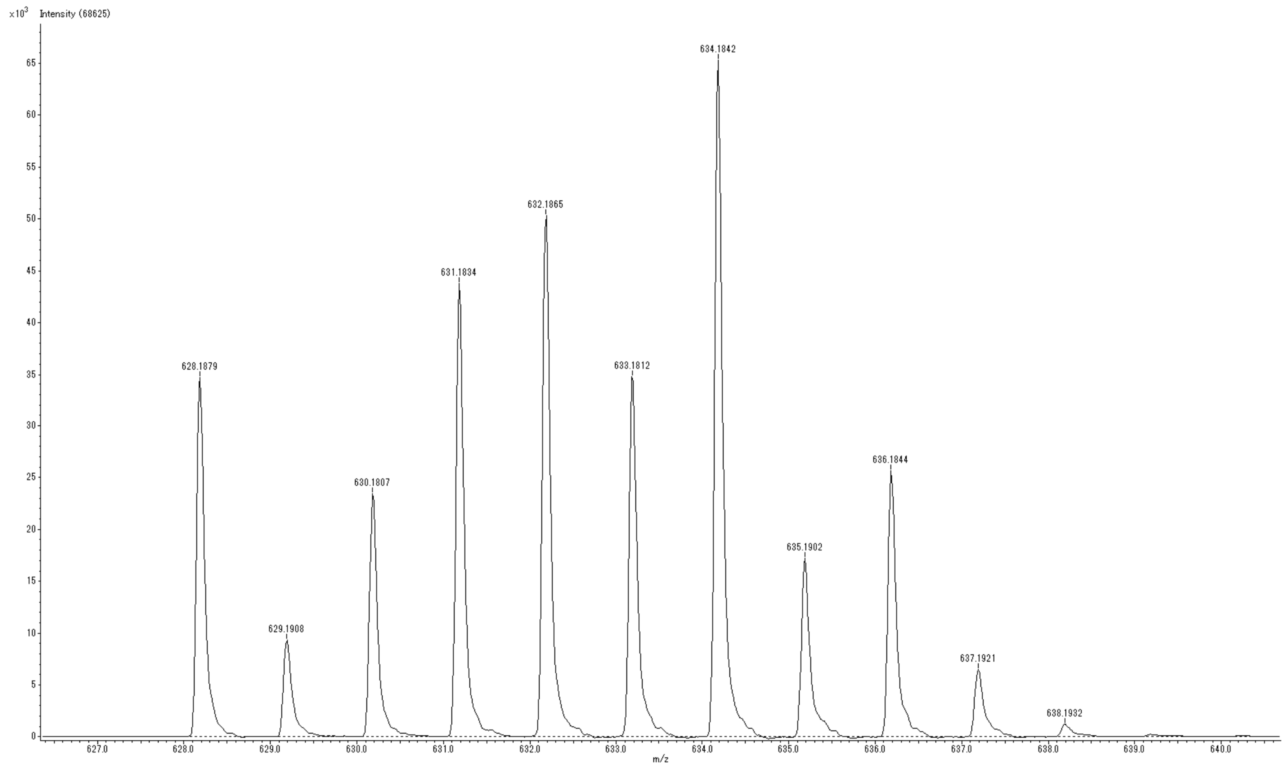

Supplementary Figure 3. ESI-TOF-MS spectrum of the residue obtained from the reduction of **2-^{15}N** with SmI_2 under $^{14}N_2$.

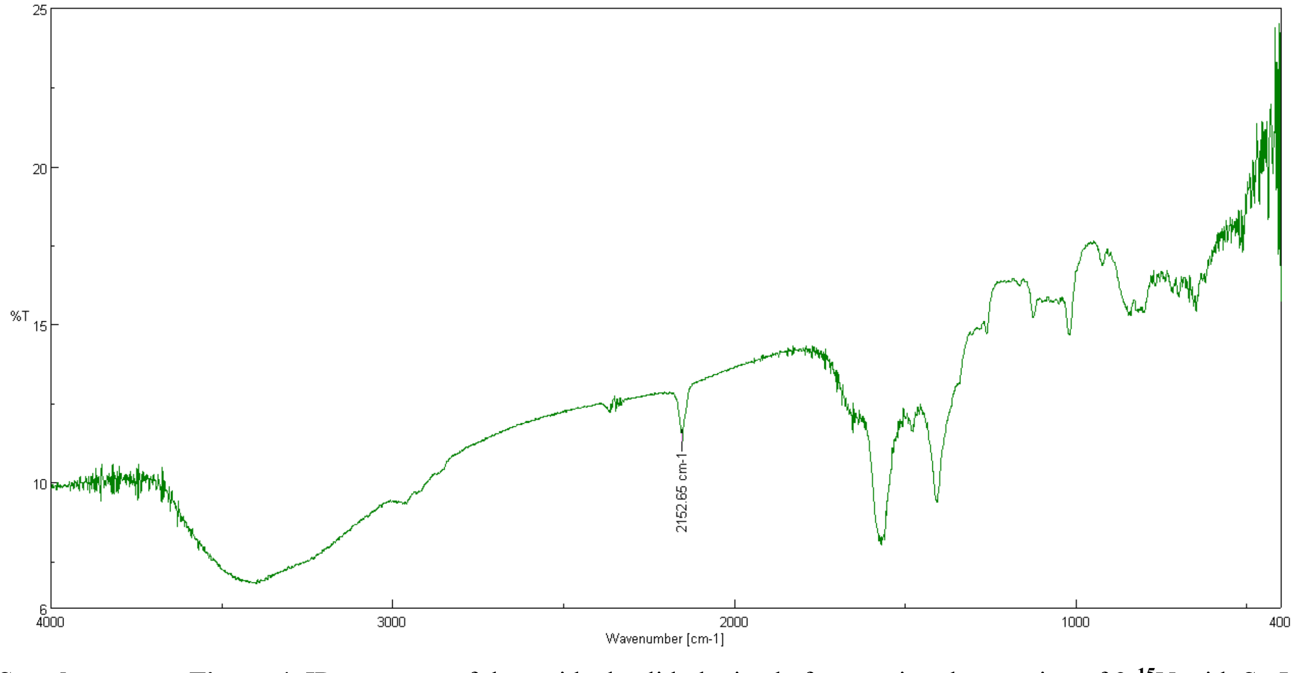

Supplementary Figure 4. IR spectrum of the residual solid obtained after treating the reaction of **2-^{15}N** with SmI_2 under $^{14}N_2$ with KO^tBu.

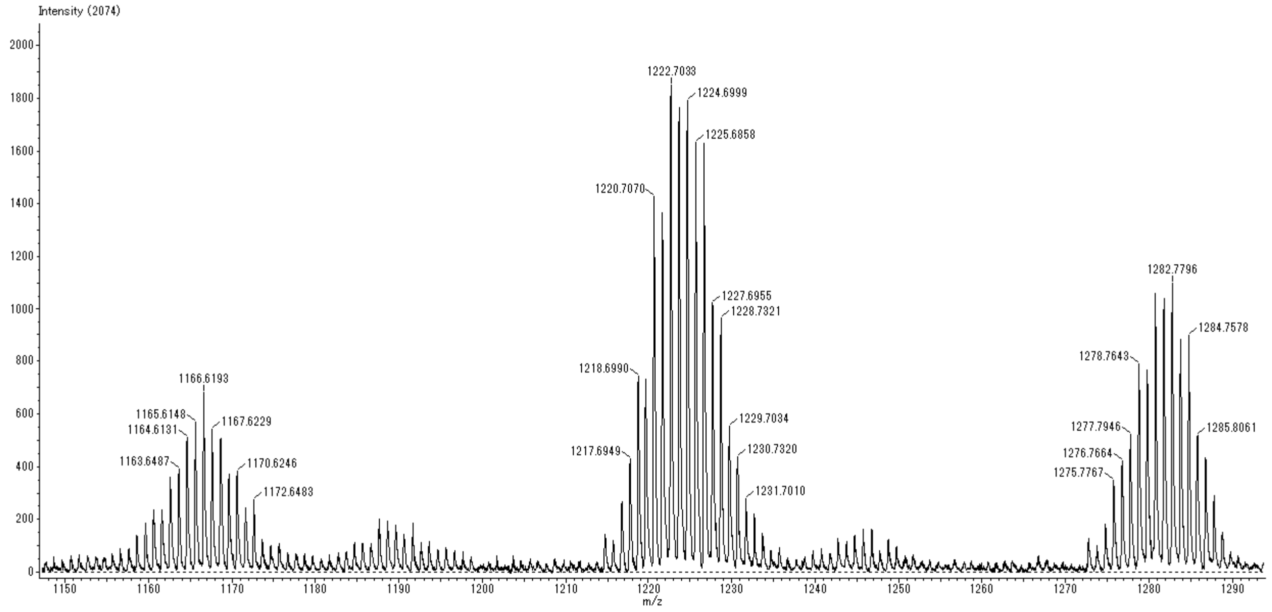

Supplementary Figure 5. ESI-TOF-MS spectrum of the residue obtained from the reaction of **4b** with $CoCp^*_2$ in the presence of NaI under N_2.

Cyclic Voltammograms.

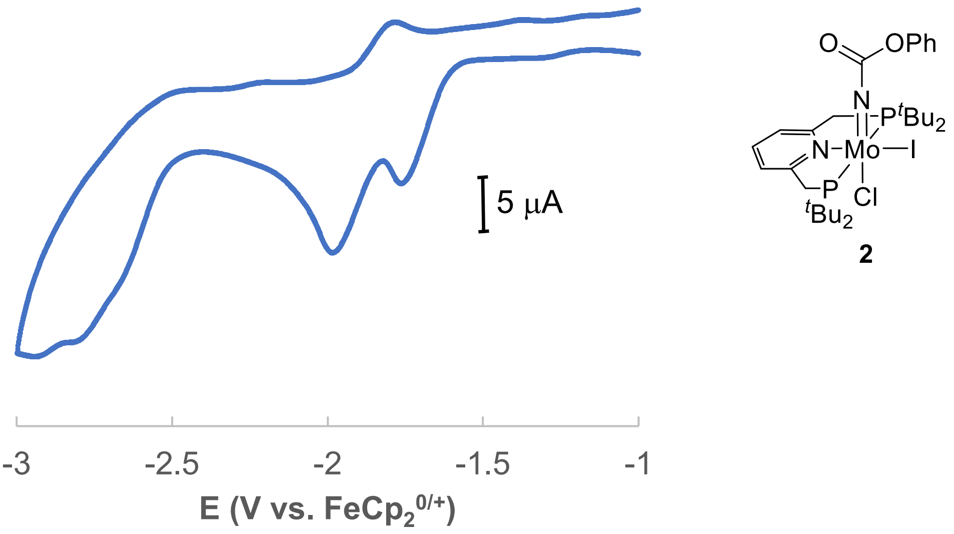

Supplementary Figure 6. Cyclic voltammograms of carbamate complex **2**.

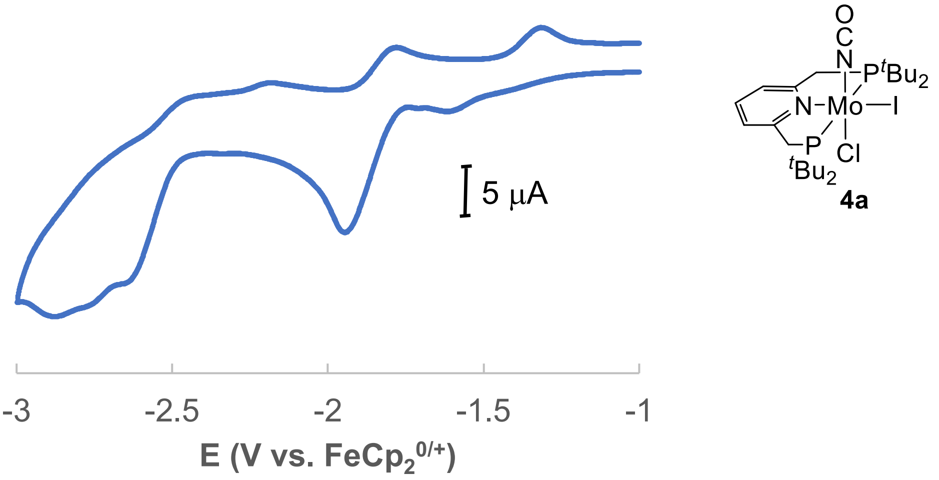

Supplementary Figure 7. Cyclic voltammograms of cyanate complex **4a**.

NMR Spectra.

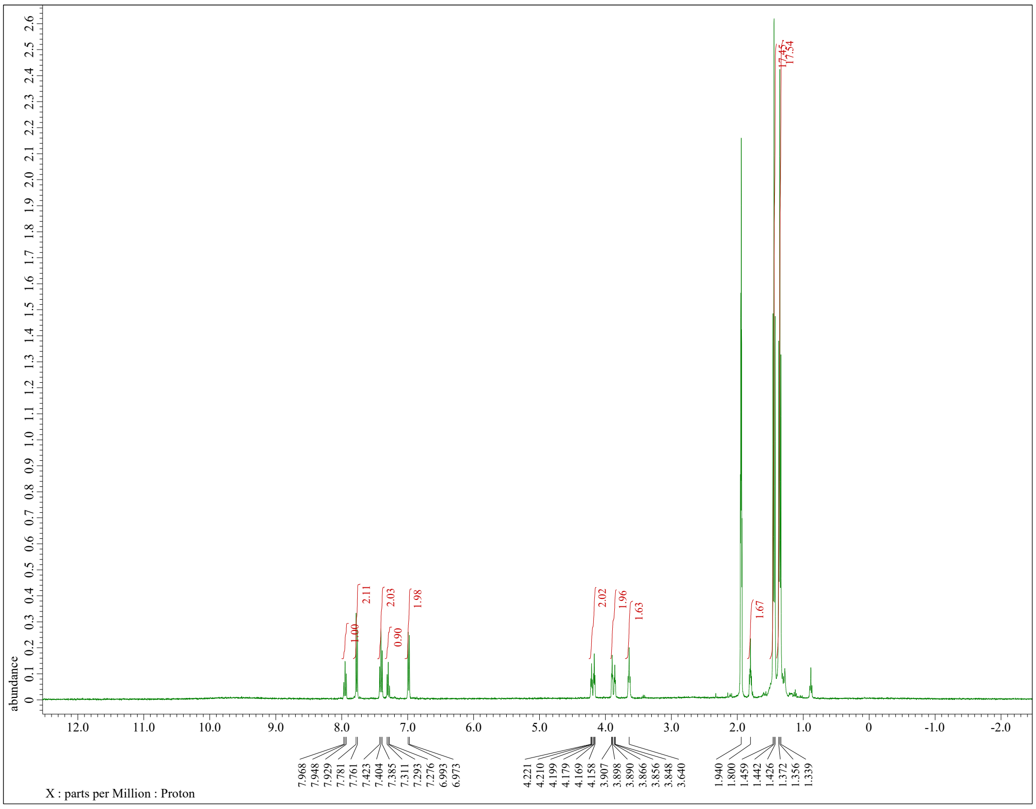

Supplementary Figure 8. ^{1}H NMR spectrum of **2** in CD_3CN.

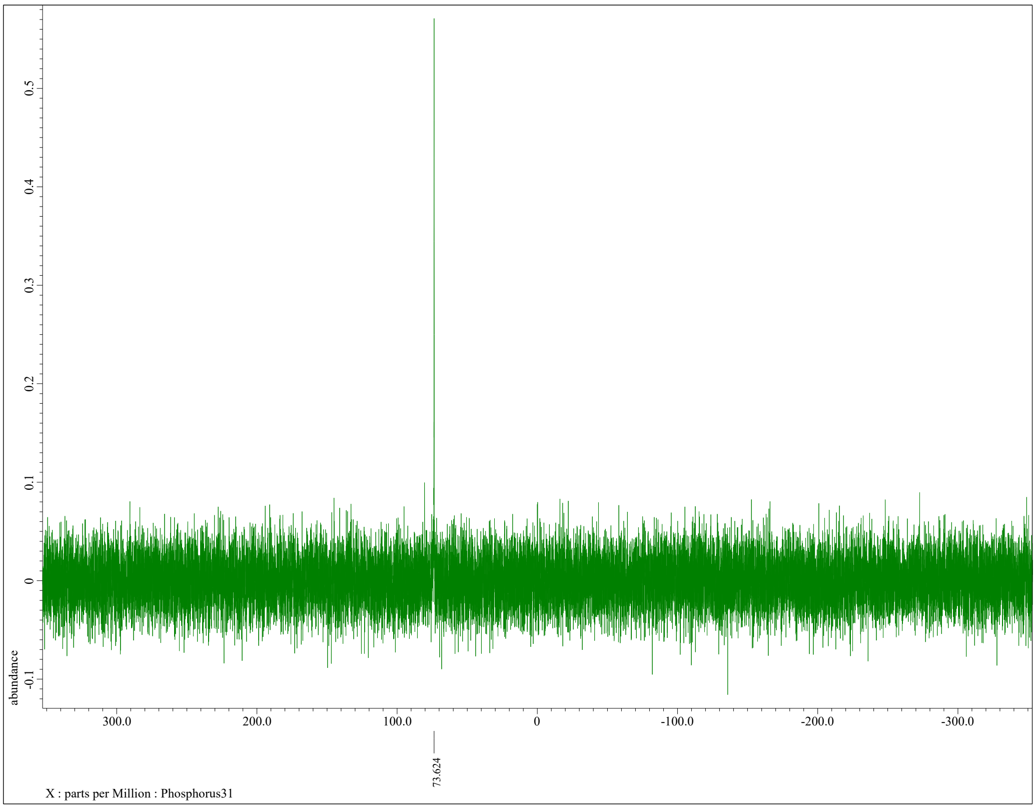

Supplementary Figure 9. $^{31}P\{^{1}H\}$ NMR spectrum of **2** in CD_3CN.

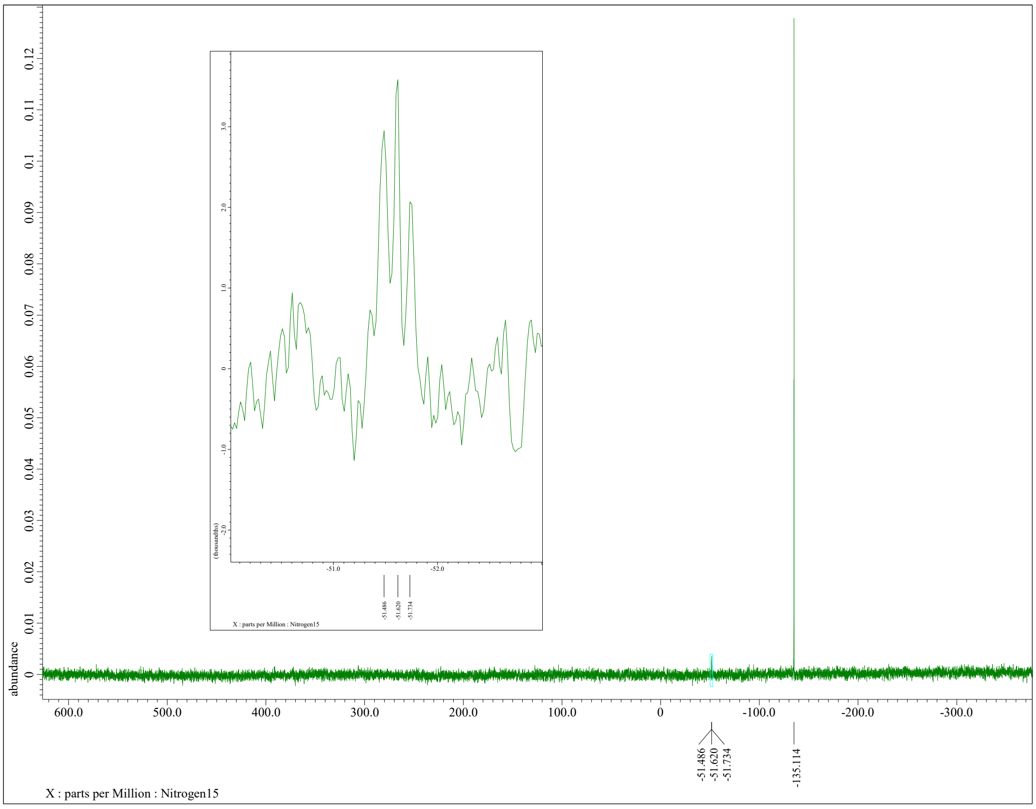

Supplementary Figure 10. $^{15}N\{^{1}H\}$ NMR spectrum of **2-^{15}N** in CD_3CN.

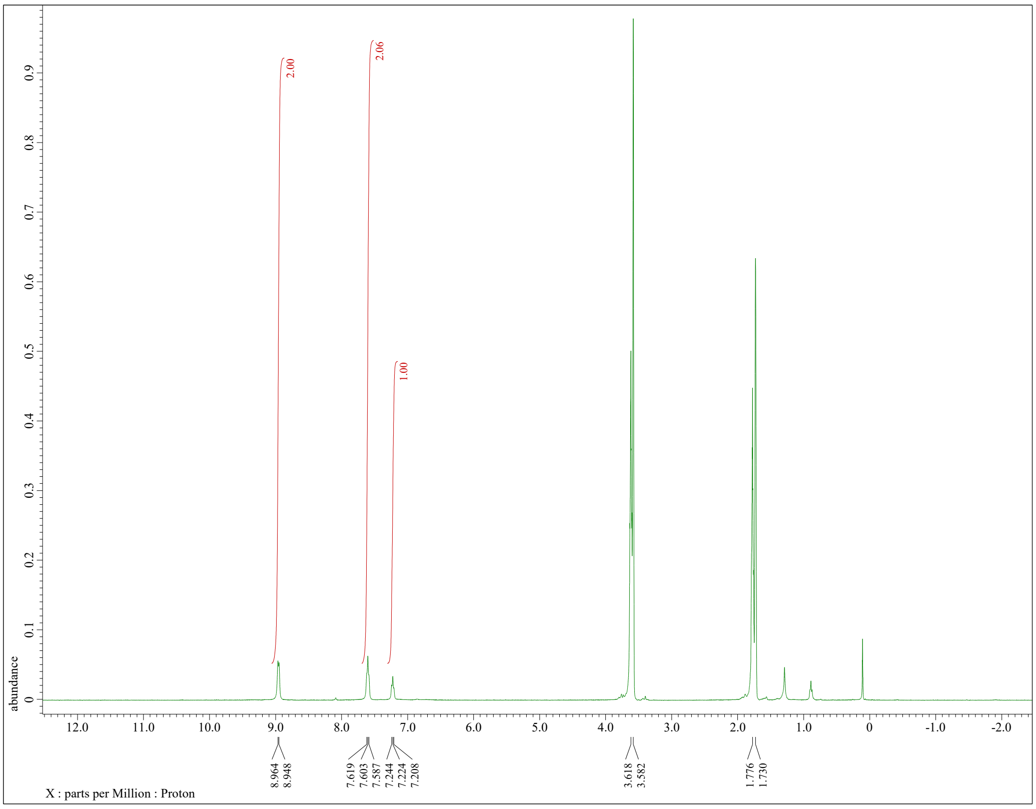

Supplementary Figure 11. ^{1}H NMR spectrum of **3a** in THF-d_8.

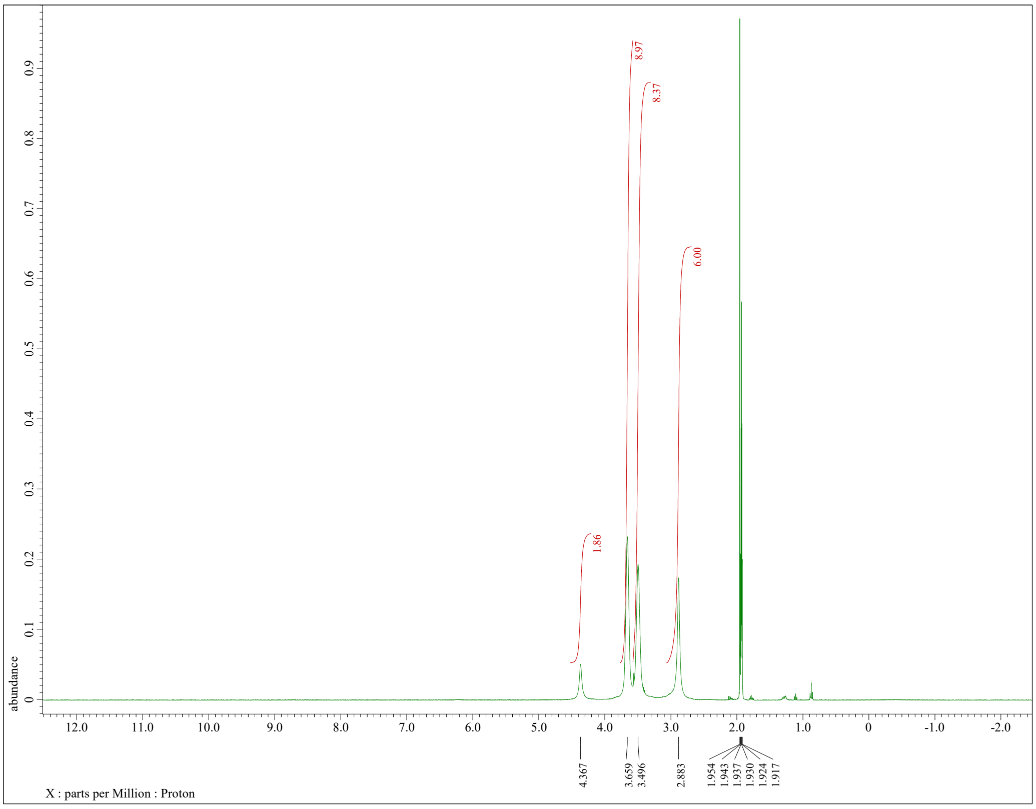

Supplementary Figure 12. ^{1}H NMR spectrum of **3b** in CD_3CN.

IR Spectra.

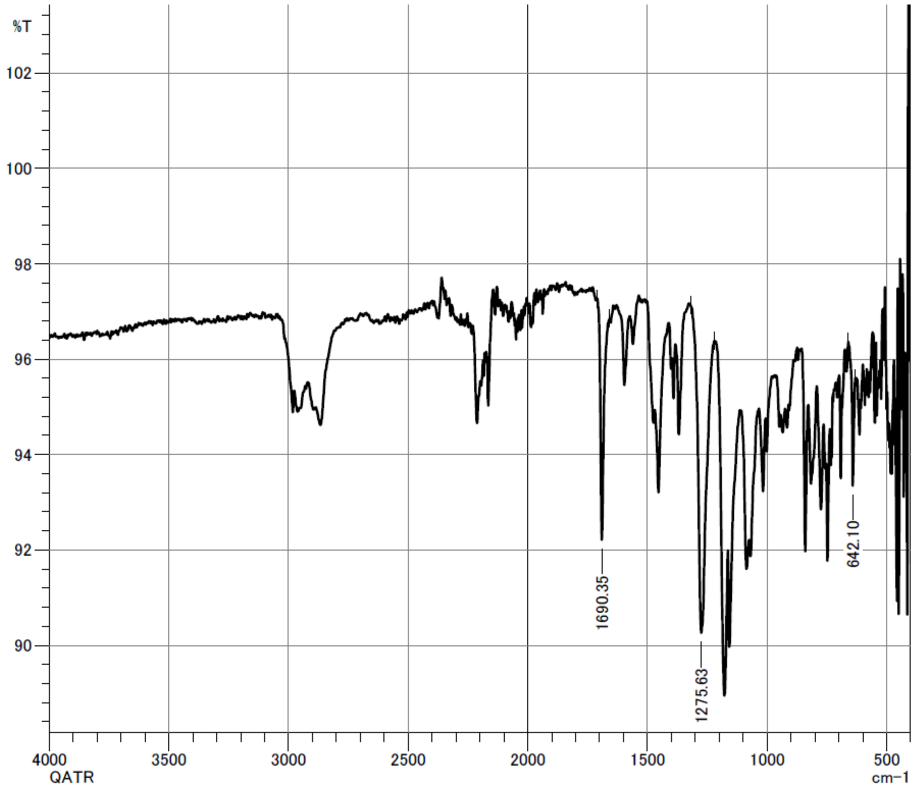

Supplementary Figure 13. ATR-IR spectrum of **2**.

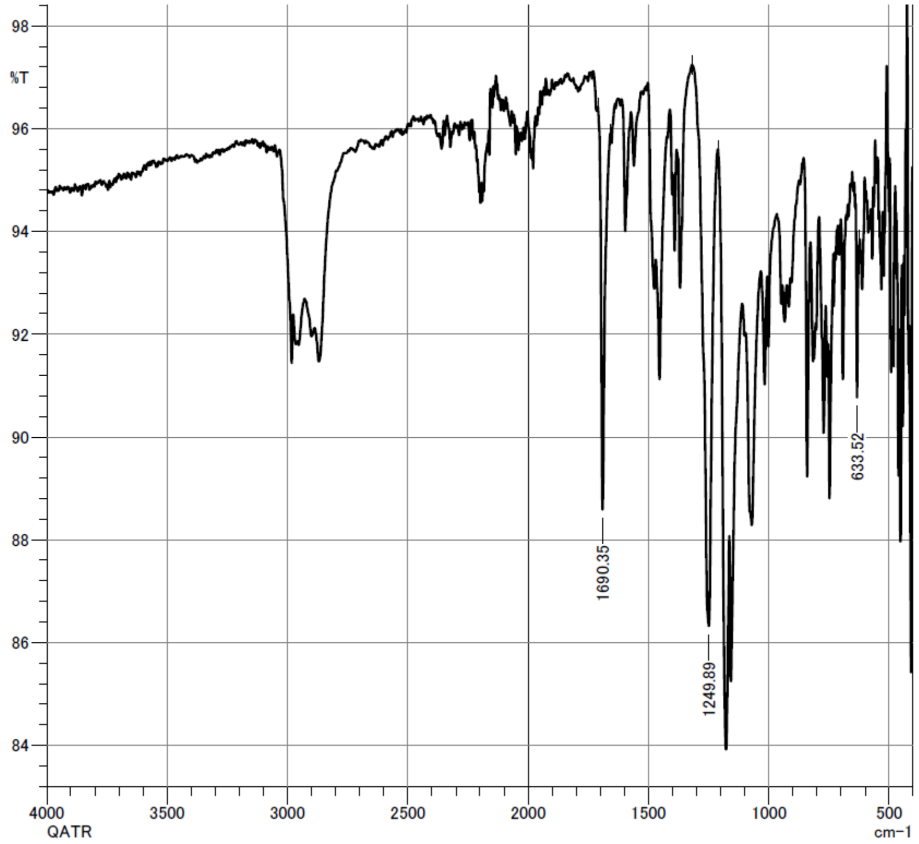

Supplementary Figure 14. ATR-IR spectrum of **2-^{15}N**.

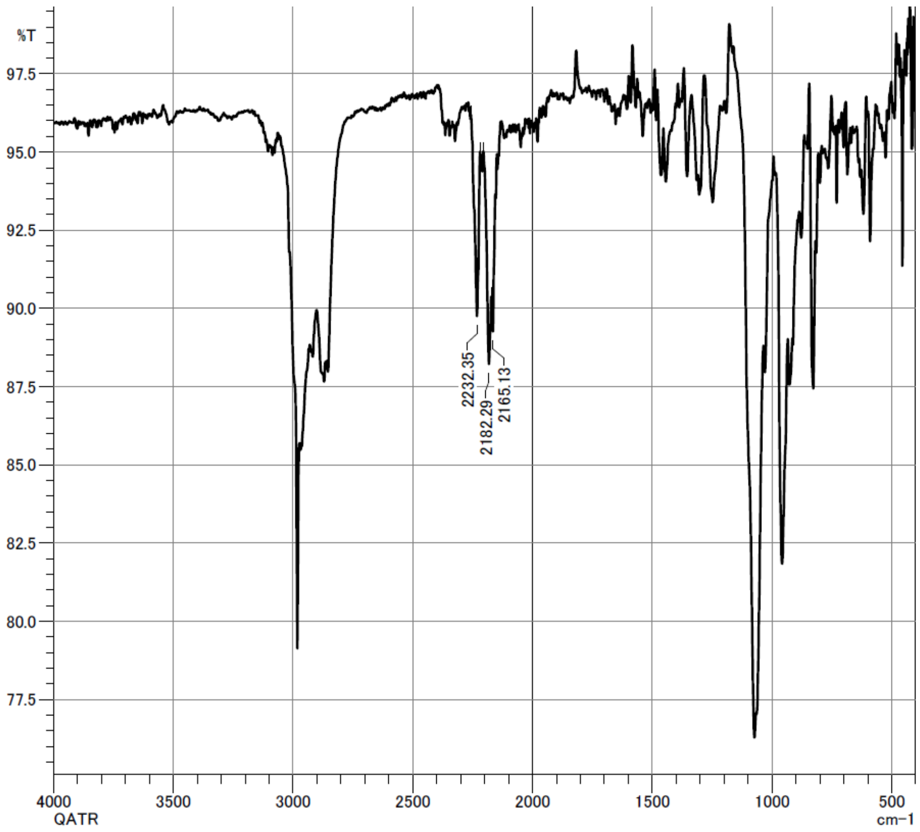

Supplementary Figure 15. ATR-IR spectrum of **3b**.

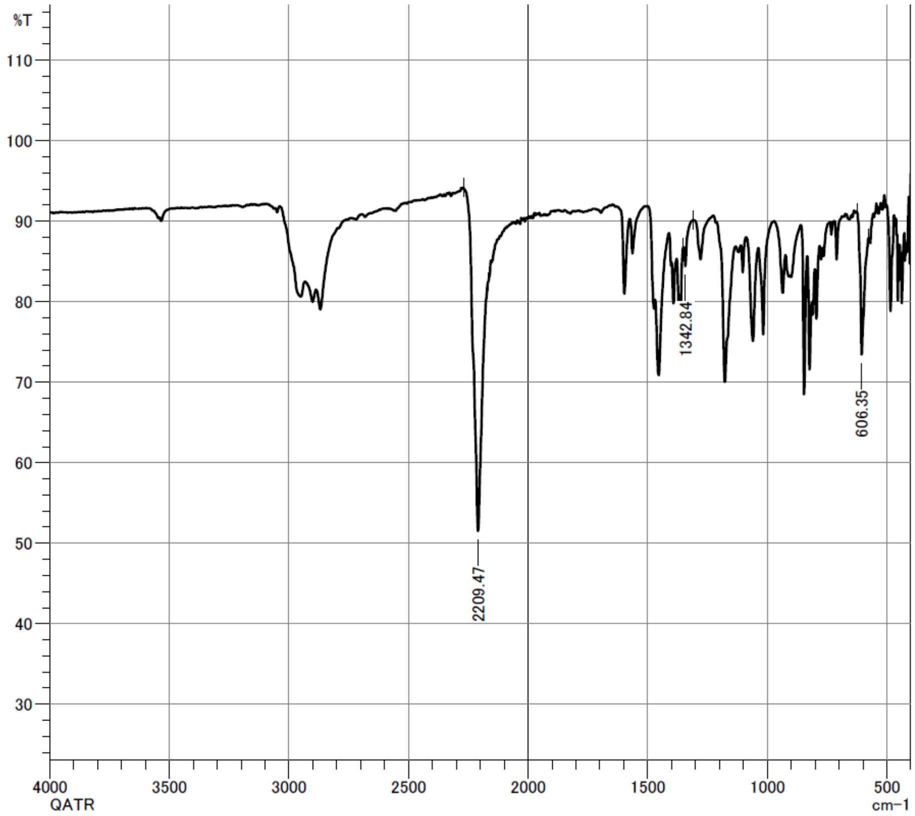

Supplementary Figure 16. ATR-IR spectrum of **4a**.

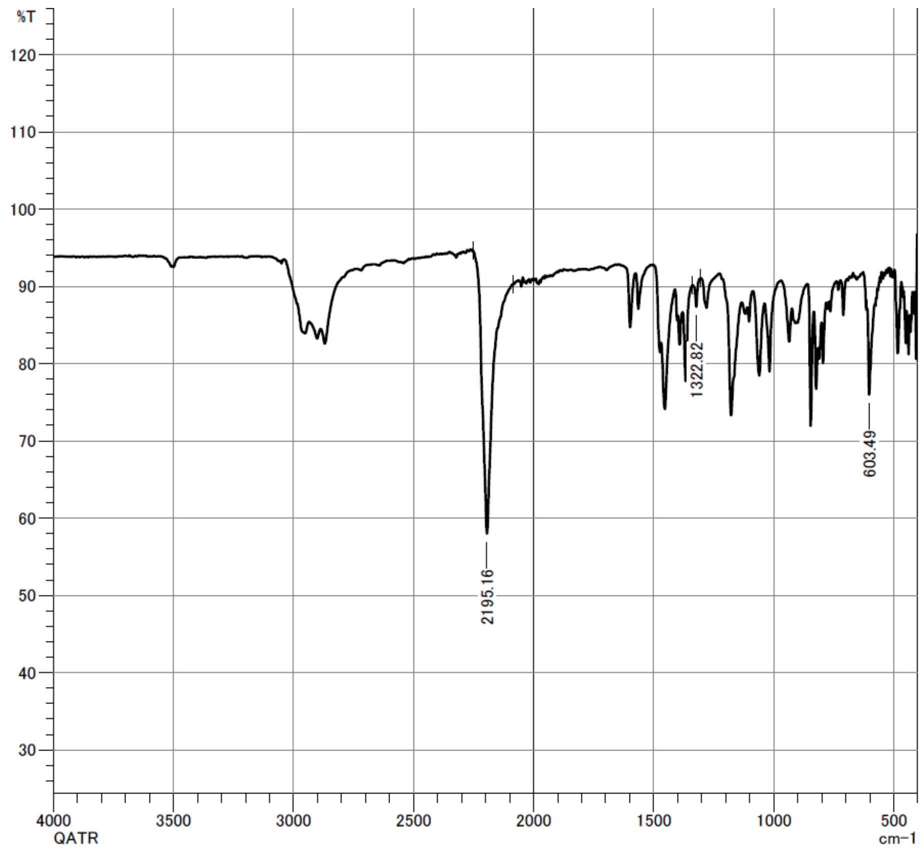

Supplementary Figure 17. ATR-IR spectrum of **4a-^{15}N**.

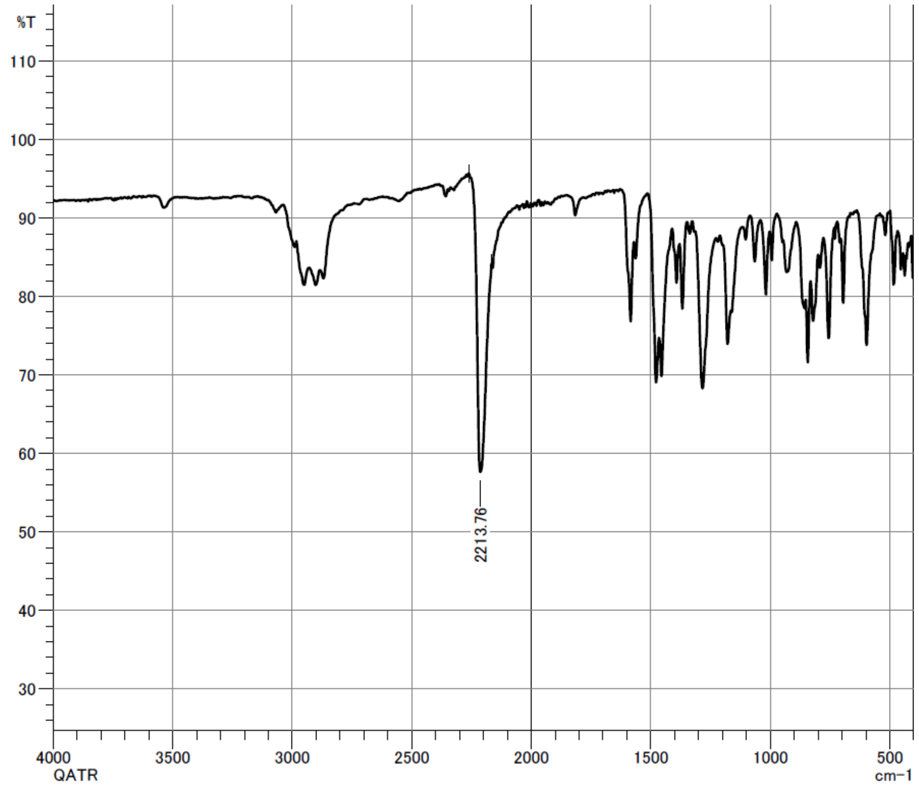

Supplementary Figure 18. ATR-IR spectrum of **4b**.

X-ray Crystal Structures

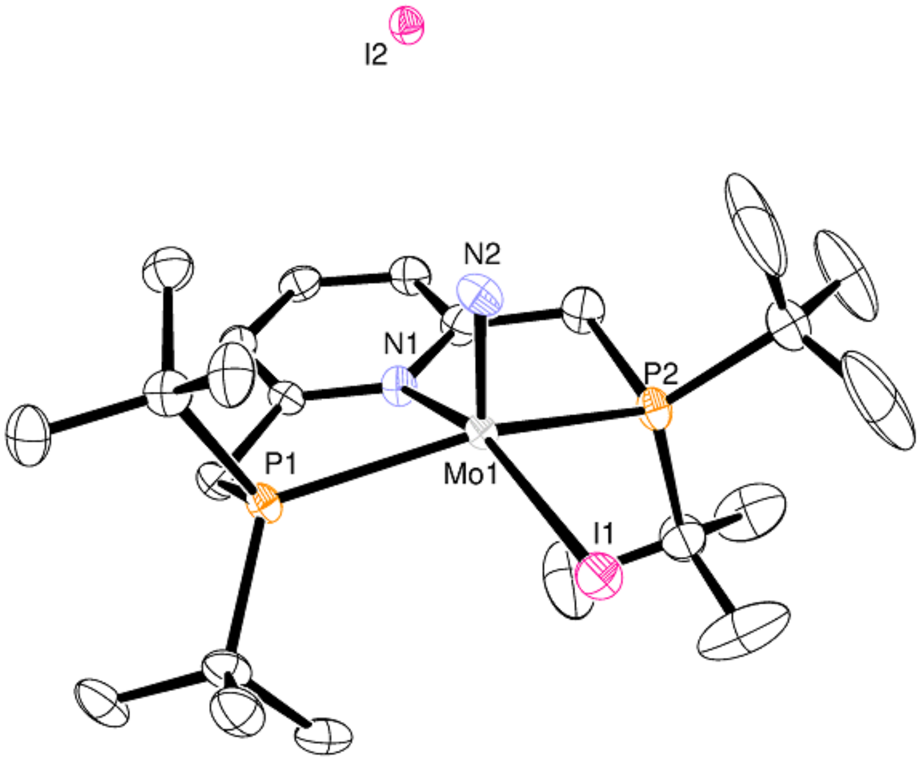

Supplementary Figure 19. Molecular structure of **[1][I]**. Thermal ellipsoids are shown at the 50% probability level, and hydrogen atoms are omitted for clarity. Selected bond lengths (Å) and angles (deg): Mo1–N1 2.229(3), Mo1–N2 1.644(3), Mo1–P1 2.5517(11), Mo1–P2 2.5360(11), Mo1–I1 2.7121(5); P1–Mo1–P2 150.90(4), N1–Mo1–I1 159.28(8), N2–Mo1–I1 102.47(12), N1–Mo1–N2 98.21(14).

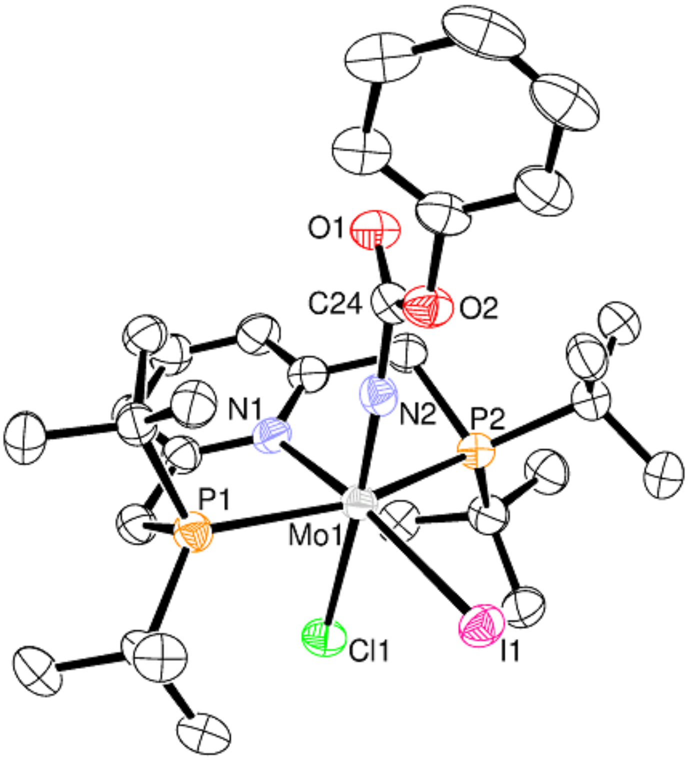

Supplementary Figure 20. Molecular structure of **2**. Thermal ellipsoids are shown at the 50% probability level, and hydrogen atoms are omitted for clarity. Selected bond lengths (Å) and angles (deg): Mo1–N1 2.187(3), Mo1–N2 1.766(3), Mo1–P1 2.5558(10), Mo1–P2 2.5420(10), Mo1–Cl1 2.4469(10), Mo1–I1 2.8390(3), N2–C24 1.358(4), O1–C24 1.220(4), O2–C24 1.364(4); P1–Mo1–P2 157.55(3), Mo1–N2–C24 173.4(2), N2–C24–O1 124.9(3), N2–C24–O2 111.5(3), O1–C24–O2 123.6(3).

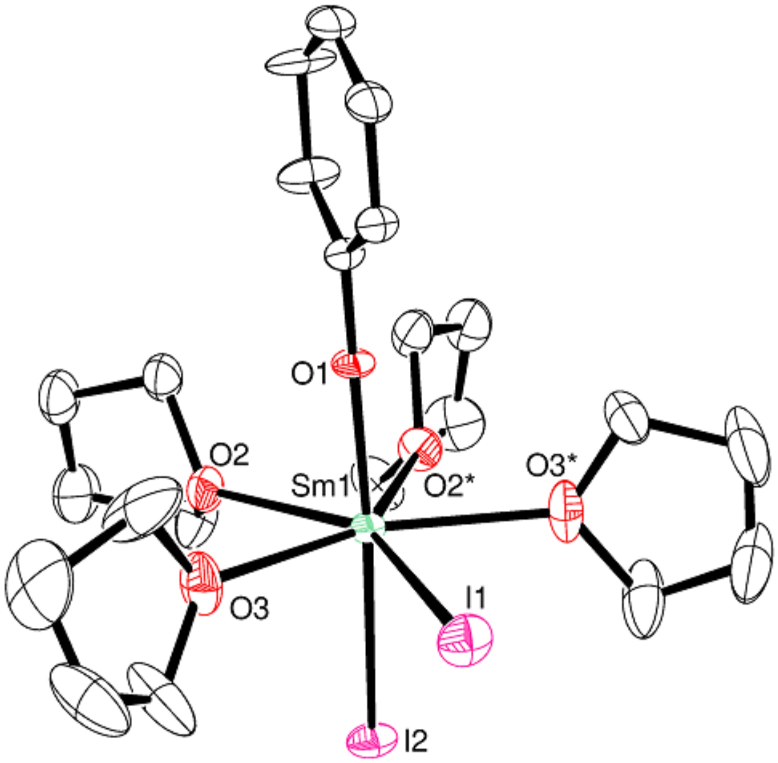

Supplementary Figure 21. Molecular structure of one of the two crystallographically independent molecules of **3a**. Thermal ellipsoids are shown at the 50% probability level, and ones of the two disorders of the CH_2 groups bound to the O2 atm as well as hydrogen atoms and the I3 atom are omitted for clarity. Selected bond lengths (Å) and angles (deg): Sm1–I1 3.1683(8), Sm1–I2 3.1016(16), Sm1–O1 2.116(8), Sm1–O2 2.472(5), Sm1–O3 2.428(5); I1–Sm1–I2 88.77(3), I1–Sm1–O1 92.86(17), I1–Sm1–O3 75.09(12), O2–Sm1–O3 69.40(16).

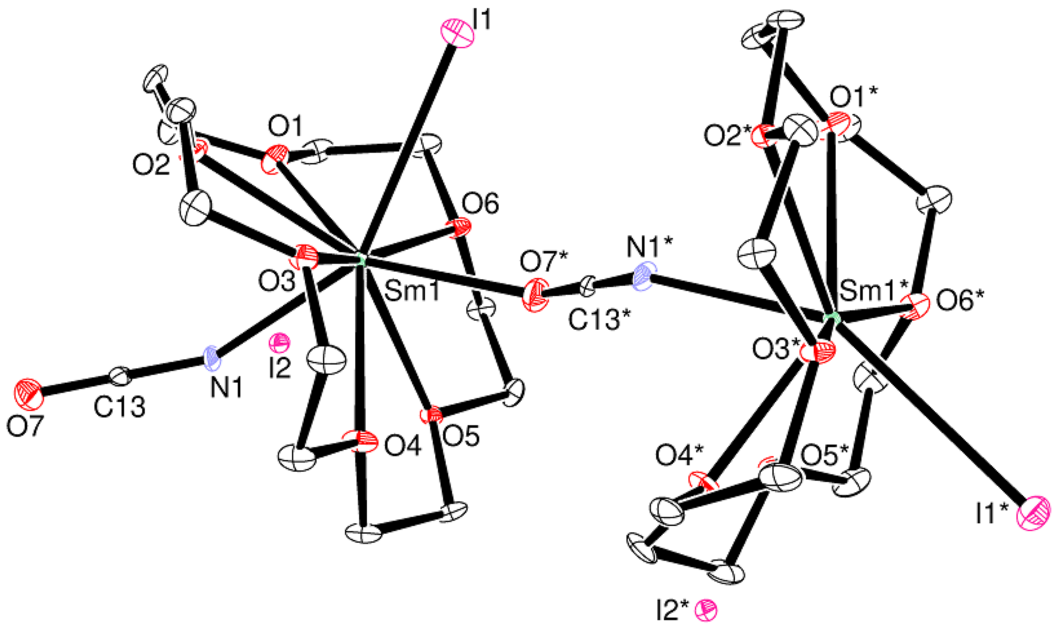

Supplementary Figure 22. Molecular structure of **3b**. Thermal ellipsoids are shown at the 50% probability level, and hydrogen atoms are omitted for clarity. Selected bond lengths (Å) and angles (deg): Sm1–I1 3.1751(5), Sm1–N1 2.392(6), Sm1–O1 2.567(4), Sm1–O2 2.499(5), Sm1–O3 2.542(4), Sm1–O4 2.578(4), Sm1–O5 2.577(4), Sm1–O6 2.578(5), Sm1–O7* 2.410(5), N1–C13 1.215(9), O7–C13 1.189(9); I1–Sm1–N1 147.89(13), I1–Sm1–O7* 74.50(13), N1–Sm1–O7* 137.08(19), Sm1–N1–C13 142.7(5), N1–C13–O7* 179.8(8).

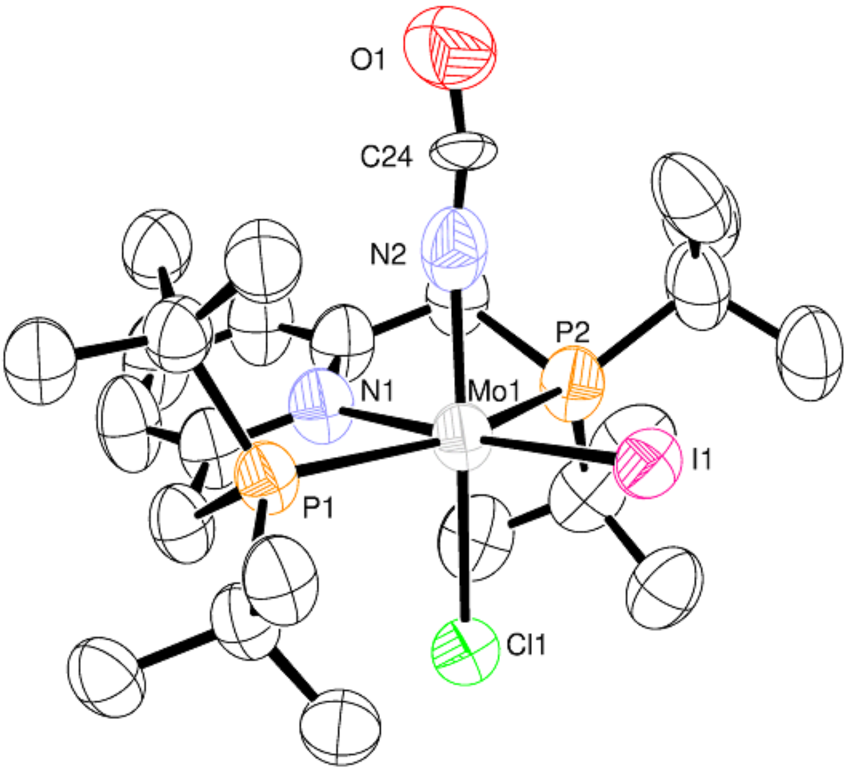

Supplementary Figure 23. Molecular structure of one of the two crystallographically independent molecules of **4a**. Thermal ellipsoids are shown at the 50% probability level, and hydrogen atoms are omitted for clarity. Selected bond lengths (Å) and angles (deg): Mo1–N1 2.193(4), Mo1–N2 2.104(5), Mo1–P1 2.5864(12), Mo1–P2 2.5973(14), Mo1–Cl1 2.5011(12), Mo1–I1 2.7388(5), N2–C24 1.125(6), O1–C24 1.212(6); P1–Mo1–P2 156.89(4), Mo1–N2–C24 165.8(4), N2–C24–O1 166.2(5).

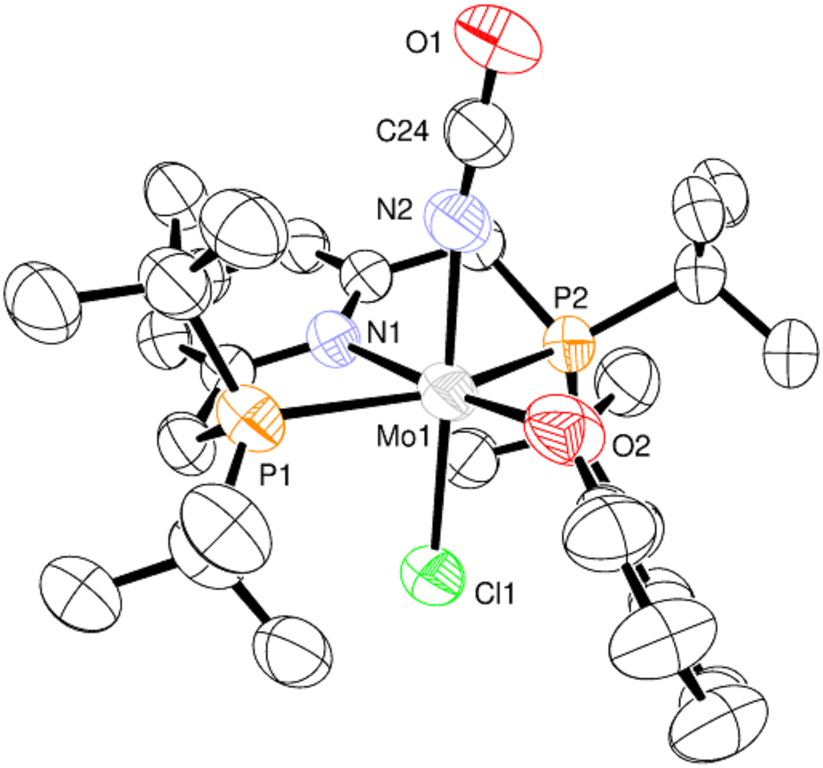

Supplementary Figure 24. Molecular structure of **4b**. Thermal ellipsoids are shown at the 50% probability level, and hydrogen atoms are omitted for clarity. Selected bond lengths (Å) and angles (deg): Mo1–N1 2.200(4), Mo1–N2 2.119(5), Mo1–P1 2.611(2), Mo1–P2 2.598(2), Mo1–Cl1 2.4084(15), Mo1–O2 2.057(4), N2–C24 1.152(8), O1–C24 1.186(7); P1–Mo1–P2 155.39(5), Mo1–N2–C24 165.8(5), N2–C24–O1 178.3(7).

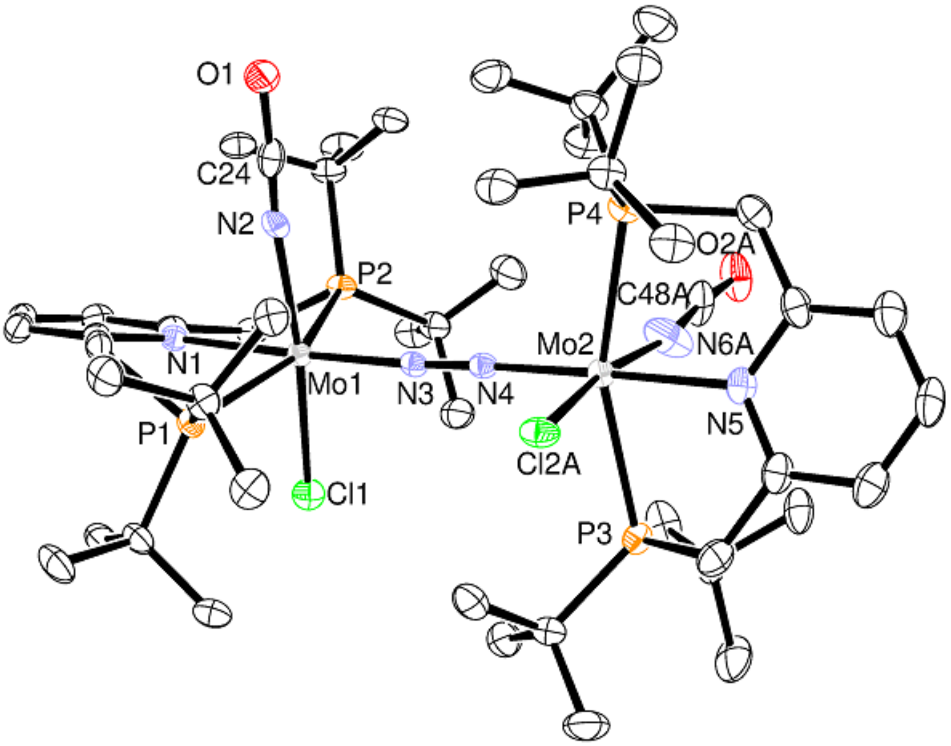

Supplementary Figure 25. Molecular structure of $[Mo(NCO)Cl(PNP)]_2(\mu\text{-}N_2)$. Thermal ellipsoids are shown at the 50% probability level, and ones of the two disorders between the Cl groups and isocyanate groups bound to the Mo2 atm as well as hydrogen atoms are omitted for clarity. Selected bond lengths (Å) and angles (deg): Mo1–N1 2.202(3), Mo1–N2 2.166(3), Mo1–N3 1.937(3), Mo1–P1 2.5666(9), Mo1–P2 2.5484(9), Mo1–Cl1 2.4483(11), N2–C24 1.051(5), O1–C24 1.257(6), Mo2–N4 1.932(3), Mo2–N5 2.191(3), Mo2–N6A 2.04(2), Mo2–P3 2.5386(10), Mo2–P4 2.5604(10), Mo2–Cl2A 2.471(4), N6A–C48A 1.252(19), O2A–C48A 1.150(12), N3–N4 1.161(4); P1–Mo1–P2 156.80(3), Mo1–N3–N4 176.0(3), Mo1–N2–C24 173.6(3), N2–C24–O1 177.4(5), P3–Mo2–P4 158.19(4), Mo2–N4–N3 177.5(2), Mo2–N6A–C48A 169(2), N6A–C48A–O2A 172.7(16).

Optimized Structures

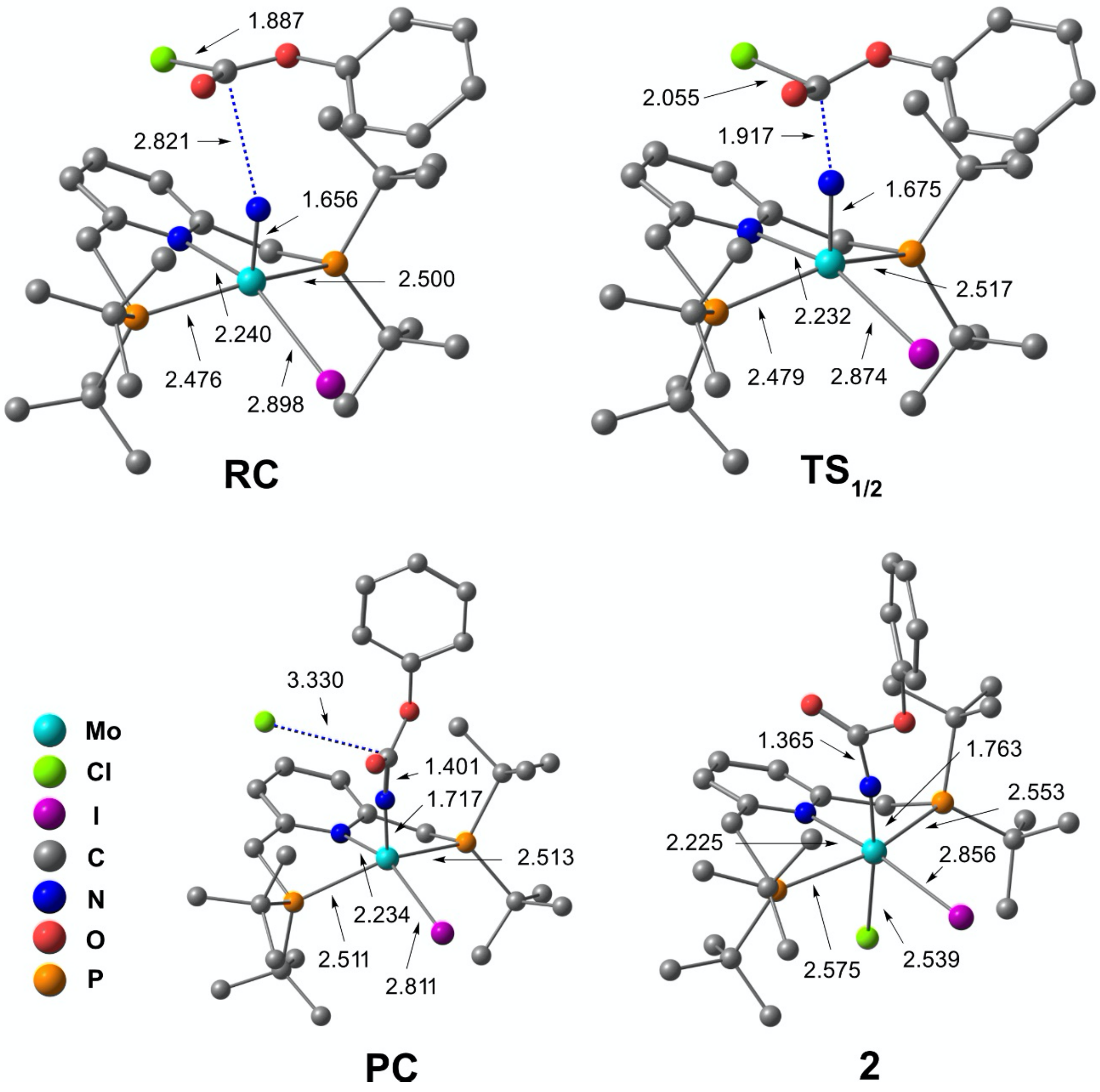

Supplementary Figure 26. Optimized structures of **RC**, **TS$_{1/2}$**, **PC**, and **2** in the closed-shell singlet as the ground spin state in Figure 5a. Selected interatomic distances are presented in Å. Hydrogen atoms attached to carbon atoms are omitted for clarity.

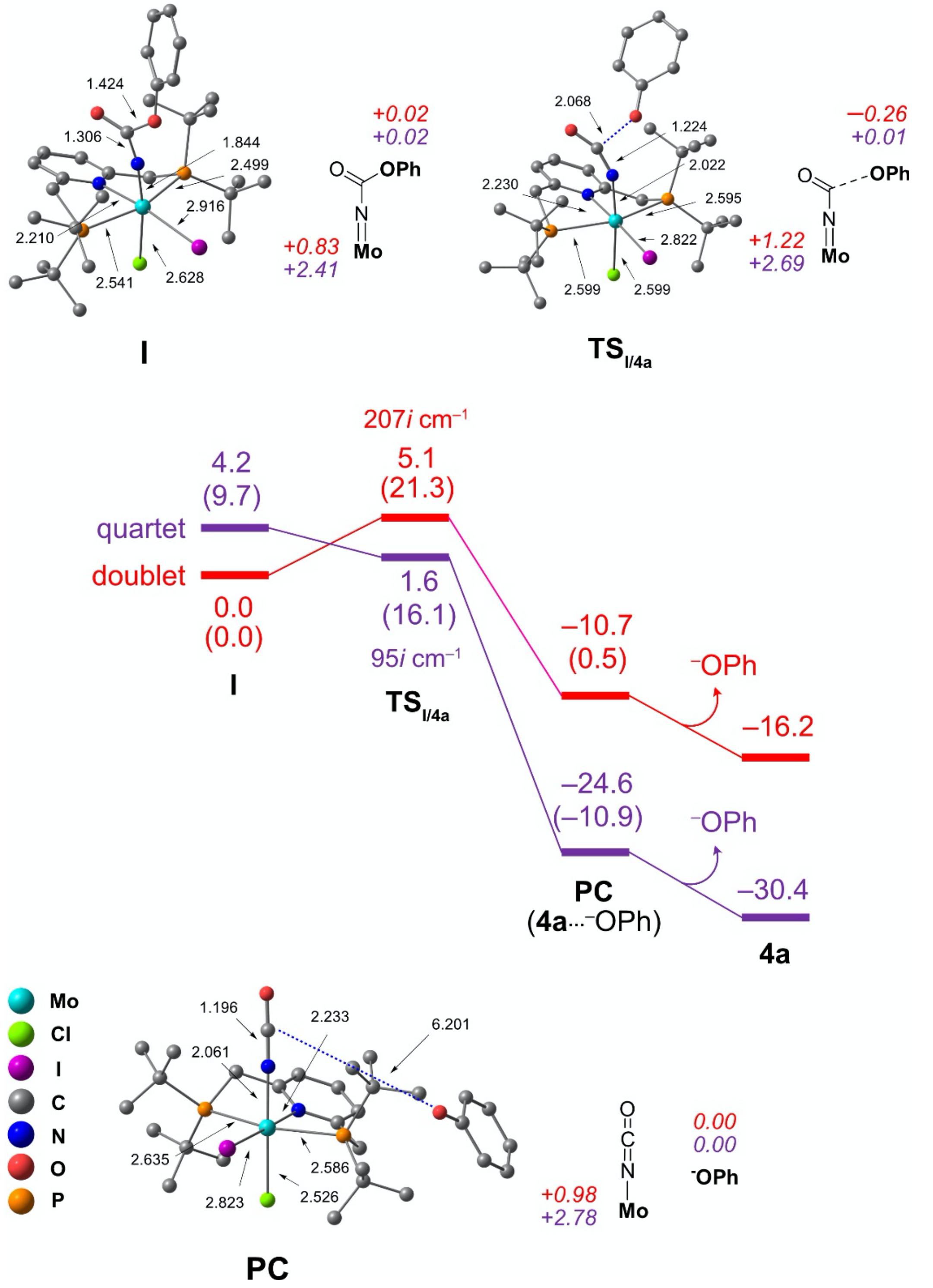

Supplementary Figure 27. Free energy profiles at 298 K (ΔG_{298} in kcal/mol) of the formation of **4a** from **I** at the doublet (red) and quartet (purple) spin states, together with optimized structures of **I**, **TS$_{\mathbf{I/4a}}$**, and **PC** in the ground spin state. Selected interatomic distances are presented in Å. Hydrogen atoms attached to carbon atoms are omitted for clarity. Energy differences relative to **I** at the doublet state *in vacuo* without thermal corrections are given in parenthesis. Mulliken spin densities assigned to the Mo atom and the leaving PhO moiety in **I**, **TS$_{\mathbf{I/4a}}$**, and **PC** are presented in italics.

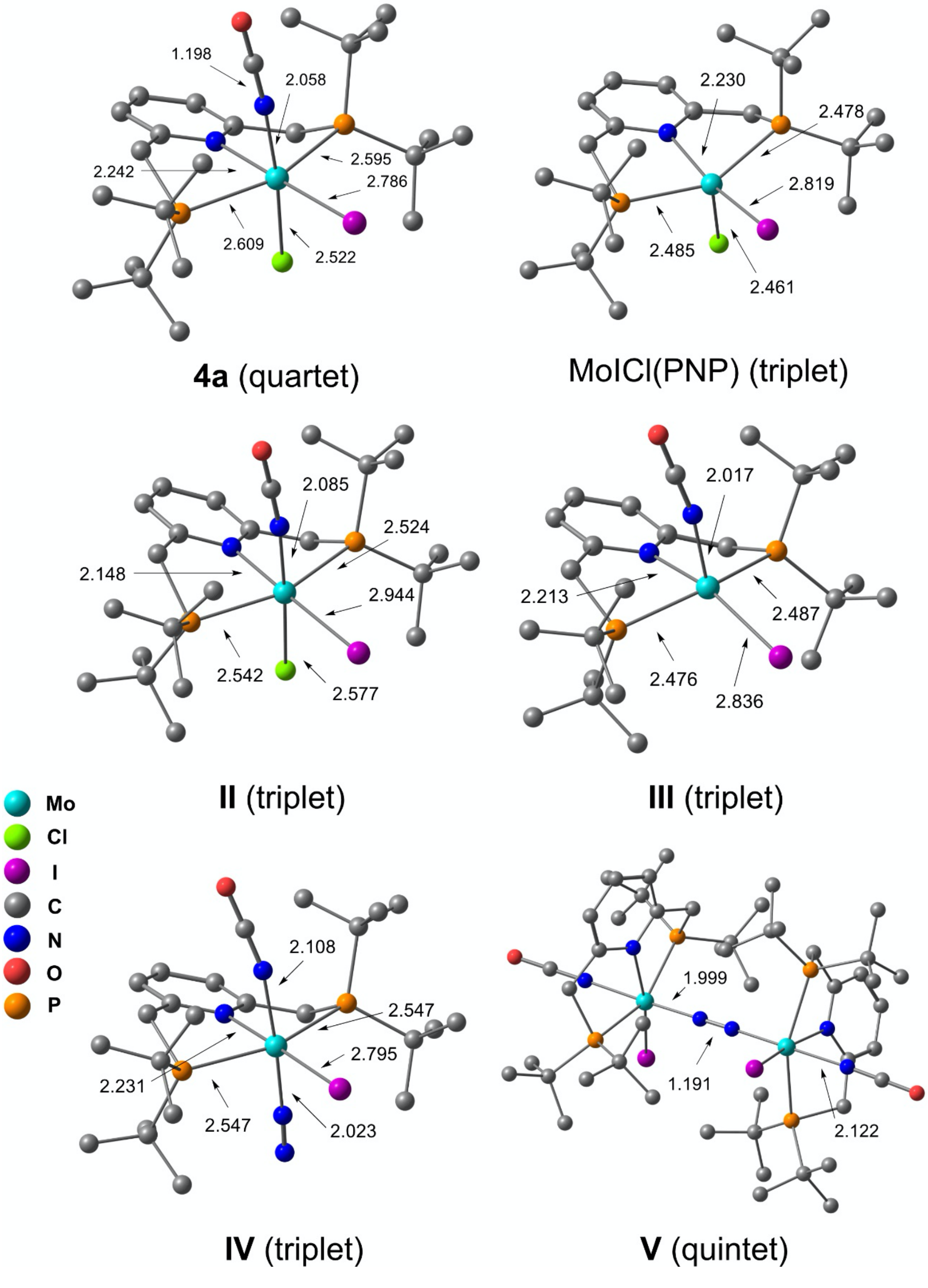

Supplementary Figure 28. Optimized structures of **4a**, MoICl(PNP) and intermediates described in Figure 6 at the ground spin state. Selected interatomic distances are presented in Å. Hydrogen atoms attached to carbon atoms are omitted for clarity.

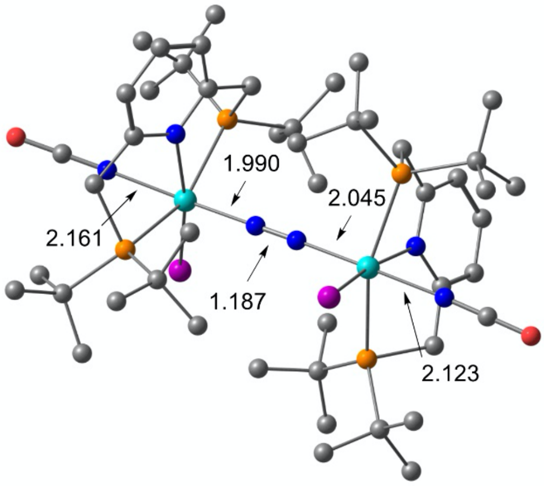

VI (quartet)

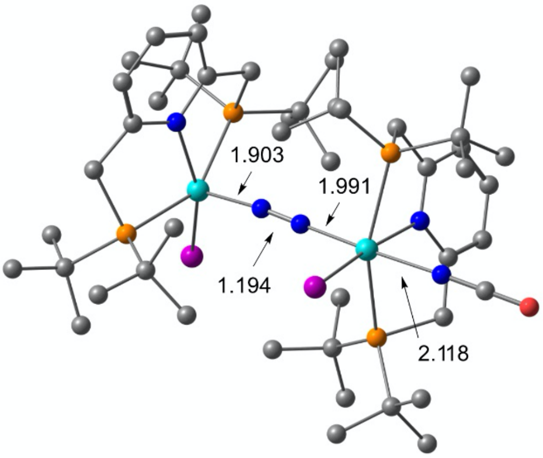

VII (quartet)

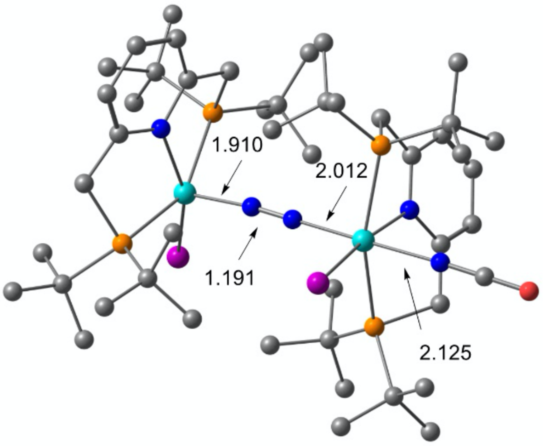

VIII (triplet)

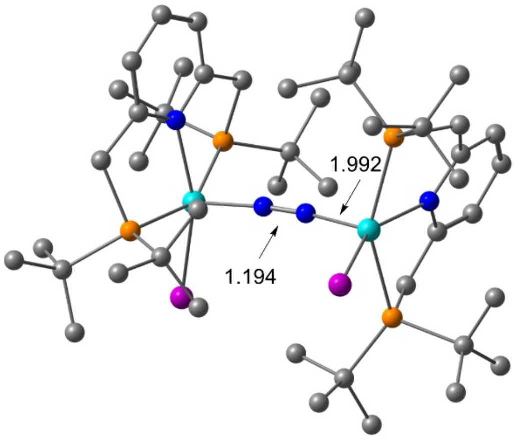

A (triplet)

Supplementary Figure 28. (continued)

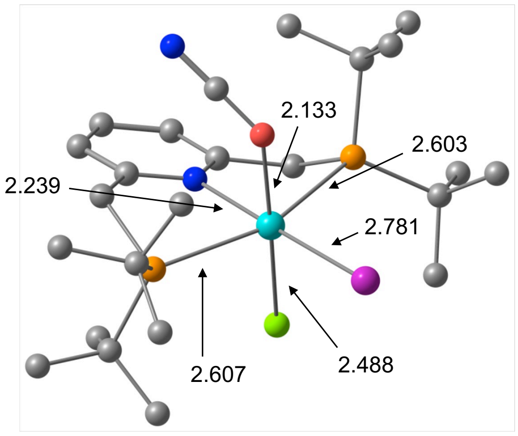

4a′ (quartet)

Supplementary Figure 29. Optimized structure of **4a′** in the ground spin state. Selected interatomic distances are presented in Å. Hydrogen atoms attached to carbon atoms are omitted for clarity.

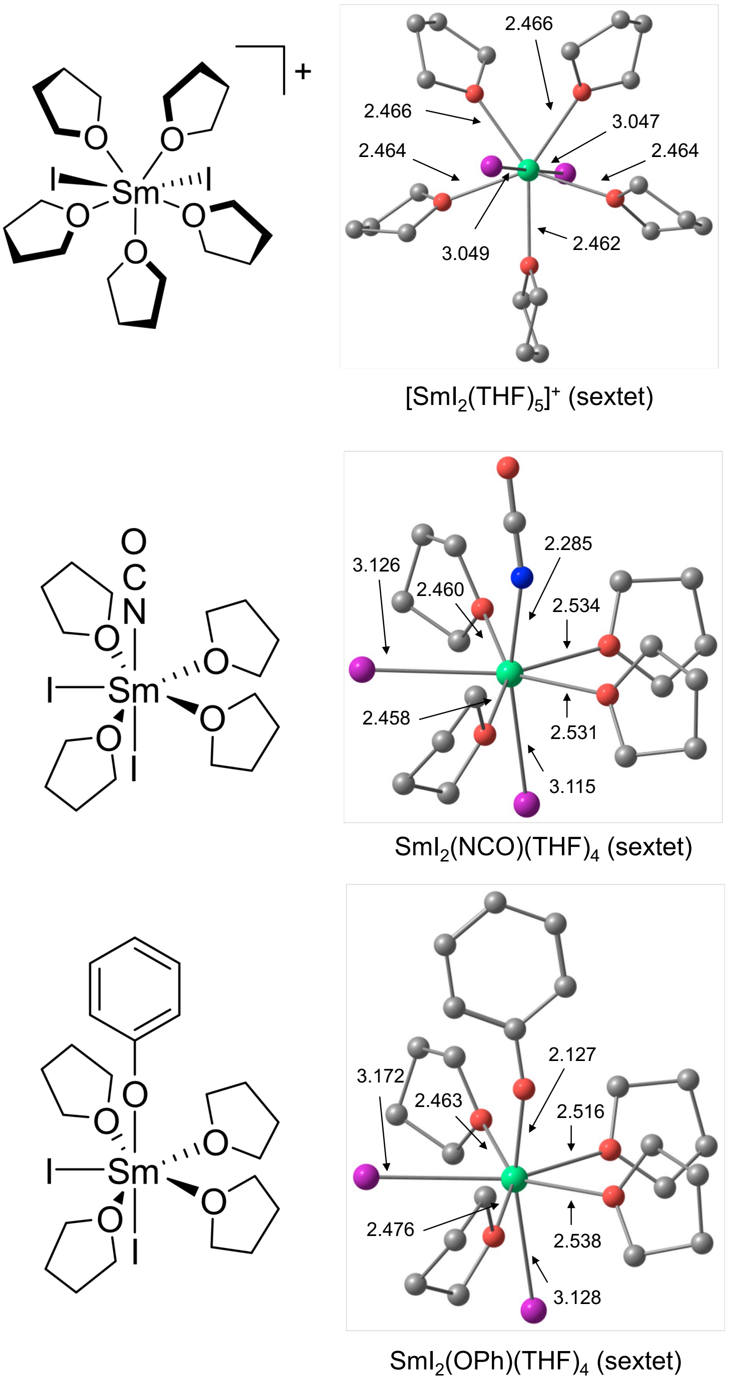

Supplementary Figure 30. Optimized structures of $[SmI_2(THF)_5]^+$, $SmI_2(NCO)(THF)_4$, and $SmI_2(OPh)(THF)_4$ in their ground spin state. Selected interatomic distances are presented in Å. Hydrogen atoms attached to carbon atoms are omitted for clarity.

Supplementary Tables.

Supplementary Table 1. Catalytic Synthesis of NCO^- from N_2

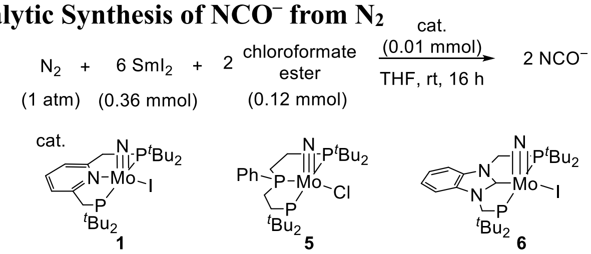

run	Catalyst	chloroformate ester	slow addition time (h)	NCO^- (equiv/Mo)
1	**1**	PhOCOCl	-	0.45
2	**5**	PhOCOCl	-	0.24
3	**6**	PhOCOCl	-	0.74
4	**1**	PhOCOI	-	0.01
5	**1**	MeOCOCl	-	0.56
6	**6**	MeOCOCl	-	0.67
7	**6**	PhOCOCl	1	0.81
8	**6**	PhOCOCl	3	1.43 ± 0.12[a]
9	**6**	PhOCOCl	6	0.86
10[b]	**6**	PhOCOCl	3	0.80
11[c]	**6**	PhOCOCl	3	1.06
12	**6**	MeOCOCl	3	2.51 ± 0.20[d]
13	**6**	EtOCOCl	3	0.93
14	**6**	^{i}PrCOCl	3	0.54
15	-[e]	MeOCOCl	3	0.0
16[f]	**6**	MeOCOCl	3	0.18
17	**6**	-[g]	-	0.0
18[h]	**6**	MeOCOCl	3	0.79
19	**6**	MeOCOCl	1	1.69
20	**6**	MeOCOCl	6	3.24 ± 0.20[d]
21[i]	**6**	MeOCOCl	6	1.89
22[j]	**6**	MeOCOCl	6	1.13
23[k]	**6**	MeOCOCl	12	8.99 ± 0.15[a]

[a]Data were mean of two individual experiments with error bars representing s.d.
[b]THF solution (1 mL) containing chloroformate ester was used.
[c]THF solution (1 mL) containing chloroformate ester was used
[d]Data were mean of three individual experiments with error bars representing s.d.
[e]Without catalyst.
[f]Without SmI_2.
[g]Without chloroformate ester.
[h]Under Ar atmosphere.
[i]Catalyst (0.005 mmol) was used.
[j]SmI_2 (0.72 mmol) and chloroformate ester (0.24 mmol) were used.
[k]Chloroformate ester (0.24 mmol) was used.

Supplementary Table 2. X-Ray Crystallographic Data for [1][I], 2, 3a

	**[1][I]**	**2**	**3a**
chemical formula	$C_{24}H_{43}I_2MoNP_2$	$C_{30}H_{48}ClIMoN_2O_2P_2$	$C_{21.52}H_{36.6}I_{2.08}O_{4.92}Sm$
CCDC number	2159843	2159844	2159845
formula weight	759.30	788.97	788.44
dimensions of crystals, mm^3	0.30 × 0.10 × 0.10	0.217 × 0.199 × 0.080	0.254 × 0.215 × 0.188
crystal color, habit	red, platelet	orange, block	colorless, block
crystal system	monoclinic	monoclinic	monoclinic
space group	$P2_1/n$	$P2_1/n$	$P2_1/m$
a, Å	8.27884(17)	15.9058(3)	11.8010(3)
b, Å	14.7529(3)	11.8848(2)	12.8875(3)
c, Å	24.2113(5)	19.2615(4)	18.6125(4)
α, deg	90	90	90
β, deg	97.5742(19)	91.9374(17)	103.290(2)
γ, deg	90	90	90
V, $Å^3$	2931.29(10)	3639.06(12)	2754.88(11)
Z	4	4	4
ρ_{calcd}, g cm^{-3}	1.720	1.440	1.901
$F(000)$	1492	1600	1509.28
μ, cm^{-1}	26.761	13.963	44.937
temperature, °C	–180	–180	–180
trans. factors range	0.823 – 1.000	0.365 – 0.894	0.388 – 0.430
no. reflections measured	29037	32340	32057
no. unique reflections	7416 (R_{int} = 0.0417)	9699 (R_{int} = 0.1022)	7366 (R_{int} = 0.0618)
no. parameters refined	285	364	370
$R1$ ($I > 2\ \sigma(I)$)[a]	0.0333	0.0511	0.0504
$wR2$ (all data)[b]	0.0843	0.1463	0.1091
GOF (all data)[c]	1.036	1.000	1.173
max diff peak / hole, e $Å^{-3}$	+1.43 / –0.73	+1.60 / –1.58	+2.42 / –1.77

[a] $R1 = \Sigma||F_o|-|F_c||/\Sigma|F_o|$. [b] $wR2 = [\Sigma w(F_o^2-F_c^2)^2/\Sigma w(F_o^2)^2]^{1/2}$, $w = 1/[\sigma^2(F_o^2) + (qP)^2 + rP]$, $P = (\mathrm{Max}(F_o^2, 0) + 2F_c^2)/3$ [q = 0.0363 (**[1][I]**), 0.0812 (**2**), 0.0002 (**3a**); r = 6.8249 (**[1][I]**), 0 (**2**), 26.3334 (**3a**)]. [c] GOF = $[\Sigma w(F_o^2-F_c^2)^2/(N_o-N_{params})]^{1/2}$.

Supplementary Table 3. X-Ray Crystallographic Data for 3b·*n*CH_3CN, 4a

	**3b**·*n*CH_3CN	**4a**
chemical formula	$C_{15}H_{27}I_2N_2O_7Sm$	$C_{24}H_{43}ClIMoN_2OP_2$
CCDC number	2159846	2159847
formula weight	751.60	695.86
dimensions of crystals, mm^3	0.948 × 0.201 × 0.186	0.448 × 0.309 × 0.159
crystal color, habit	colorless, prism	orange, block
crystal system	monoclinic	monoclinic
space group	$P2_1/n$	$P2_1/n$
a, Å	10.8599(3)	20.6084(5)
b, Å	12.7472(4)	13.1535(2)
c, Å	16.7216(5)	26.9463(6)
α, deg	90	90
β, deg	90.035(3)	107.337(2)
γ, deg	90	90
V, Å^3	2314.83(12)	6972.6(3)
Z	4	8
ρ_{calcd}, g cm^{-3}	2.156	1.326
$F(000)$	1420.00	2808.00
μ, cm^{-1}	5.245	14.456
temperature, °C	–180	–180
trans. factors range	0.102 – 0.377	0.356 – 1.000
no. reflections measured	12905	64722
no. unique reflections	5423 (R_{int} = 0.0287)	17641 (R_{int} = 0.0507)
no. parameters refined	245	609
$R1$ ($I > 2\sigma(I)$)[a]	0.0413	0.0476
$wR2$ (all data)[b]	0.1100	0.1436
GOF (all data)[c]	0.924	1.034
max diff peak / hole, e Å^{-3}	+2.57 / –3.47	+1.84 / –1.80

[a] $R1 = \Sigma||F_o|-|F_c||/\Sigma|F_o|$. [b] $wR2 = [\Sigma w(F_o^2-F_c^2)^2/\Sigma w(F_o^2)^2]^{1/2}$, $w = 1/[\sigma^2(F_o^2) + (qP)^2 + rP]$, $P = (\mathrm{Max}(F_o^2, 0) + 2F_c^2)/3$ [q = 0.0571 (**3b**·*n*CH_3CN), 0.0727 (**4a**); r = 38.6318 (**3b**·*n*CH_3CN), 4.3750 (**4a**)]. [c] GOF $= [\Sigma w(F_o^2-F_c^2)^2/(N_o-N_{params})]^{1/2}$.

Supplementary Table 4. X-Ray Crystallographic Data for 4b, $[Mo(NCO)Cl(PNP)]_2(\mu\text{-}N_2)$

	**4b**	$[Mo(NCO)Cl(PNP)]_2(\mu\text{-}N_2)$
chemical formula	$C_{30}H_{48}ClMoN_2O_2P_2$	$C_{48}H_{86}Cl_2Mo_2N_6O_2P_4$
CCDC number	2159848	2159849
formula weight	662.06	1165.93
dimensions of crystals, mm^3	0.197 × 0.195 × 0.077	0.224 × 0.170 × 0.121
crystal color, habit	orange, block	brown, block
crystal system	monoclinic	triclinic
space group	$C2/c$	$P\bar{1}$
a, Å	27.2490(14)	12.3501(3)
b, Å	17.5692(5)	12.6487(2)
c, Å	17.0156(6)	20.6476(4)
α, deg	90	83.6454(16)
β, deg	105.552(5)	87.3344(17)
γ, deg	90	82.9993(17)
V, Å^3	7847.9(6)	3179.92(11)
Z	8	2
ρ_{calcd}, g cm^{-3}	1.121	1.218
$F(000)$	2776	1220
μ, cm^{-1}	5.067	6.145
temperature, °C	–180	–180
trans. factors range	0.246 – 0.962	0.885 – 1.00 0
no. reflections measured	35087	50813
no. unique reflections	10316 (R_{int} = 0.0861)	16276 (R_{int} = 0.0361)
no. parameters refined	355	637
$R1$ ($I > 2\sigma(I)$)[a]	0.0929	0.0519
$wR2$ (all data)[b]	0.1749	0.1375
GOF (all data)[c]	0.973	1.014
max diff peak / hole, e Å^{-3}	+1.00 / –0.59	+1.16 / –0.86

[a] $R1 = \Sigma||F_o|-|F_c||/\Sigma|F_o|$. [b] $wR2 = [\Sigma w(F_o^2-F_c^2)^2/\Sigma w(F_o^2)^2]^{1/2}$, $w = 1/[\sigma^2(F_o^2) + (qP)^2 + rP]$, P = (Max(F_o^2, 0) + 2 F_c^2)/3 [0 (**4b**), 0.0554 ($[Mo(NCO)Cl(PNP)]_2(\mu\text{-}N_2)$); r = 60 (**4b**), 6.1407 ($[Mo(NCO)Cl(PNP)]_2(\mu\text{-}N_2)$)]. [c] GOF = $[\Sigma w(F_o^2-F_c^2)^2/(N_o-N_{params})]^{1/2}$.

Supplementary Table 5. SCF energies (SCFE), thermal corrections at 298.15 K (TC_{298}), and single-point energies in THF (E_{solv}) calculated for all species in the present study. Units are given in Hartree.

Species (spin state)	SCFE	TC_{298}	E_{solv}
**RC** (**1**···PhOCOCl) (closed-shell singlet)	-2209.64338026	0.65814000	-2210.13805583
**TS$_{1/2}$** (closed-shell singlet)	-2209.63181845	0.66035100	-2210.13187705
**PC** (closed-shell singlet)	-2209.67107776	0.66014700	-2210.19265625
**2** (closed-shell singlet)	-2209.71127307	0.66519200	-2210.20349308
**I** (doublet)	-2209.77167367	0.66223900	-2210.31737179
**TS$_{I/4a}$** (quartet)	-2209.74596982	0.65965400	-2210.31216713
**PC** (**4a**···$^{-}$OPh) (quartet)	-2209.78907460	0.65451600	-2210.34880967
**4a** (quartet)	-1902.84017692	0.57417600	-1903.25508839
**4a′** (quartet)	-1902.81676186	0.57541500	-1903.23341137
MoICl(PNP) (triplet)	-1734.68167991	0.56203200	-1735.03748840
**II** (triplet)	-1902.89226649	0.57226300	-1903.35608131
**III** (triplet)	-1887.74452479	0.57007100	-1888.15117217
**IV** (triplet)	-1997.30653994	0.58105900	-1997.75023962
**V** (quintet)	-3885.12422350	1.18915000	-3885.97022305
**VI** (quartet)	-3885.18081777	1.18689100	-3886.06522346
**VII** (quartet)	-3716.98087752	1.17464200	-3717.76475104
**VIII** (triplet)	-3885.17872509	1.18981900	-3886.05880572
**A** (triplet)	-3548.83340078	1.16019300	-3549.55314102
$[SmI_2(THF)_5]^+$ (sextet)	-1843.40714432	0.51784600	-1843.82263937
$SmI_2(NCO)(THF)_4$ (sextet)	-1779.21475293	0.41622400	-1779.5835731
$SmI_2(OPh)(THF)_4$ (sextet)	-1918.01639467	0.49518900	-1918.41266114

Supplementary References.

1 Bain, G. A.; Berry, J. F. Diamagnetic Corrections and Pascal's Constants. *J. Chem. Educ.* **2008**, *85*, 532.

2 Arashiba, K.; Eizawa, A.; Tanaka, H.; Nakajima, K.; Yoshizawa, K.; Nishibayashi, Y. Catalytic Nitrogen Fixation via Direct Cleavage of Nitrogen–Nitrogen Triple Bond of Molecular Dinitrogen under Ambient Reaction Conditions. *Bull. Chem. Soc. Jpn.* **2017**, *90*, 1111–1118.

3 Hoffmann, H. M. R.; Iranshahi, L. Reactive Iodine Compounds. 8. Synthesis and Copper(I) Cyanide-Promoted Cyanation of Iodoformic Esters. *J. Org. Chem.* **1984**, *49*, 1174–1176.

4 Yandulov, D. V.; Schrock, R. R. Synthesis of Tungsten Complexes That Contain Hexaisopropylterphenyl-Substituted Triamidoamine Ligands, and Reactions Relevant to the Reduction of Dinitrogen to Ammonia. *Can. J. Chem.* **2005**, *83*, 341–357.

5 Weitz, I. S.; Rabinovitz, M. The Application of C_8K for Organic Synthesis: Reduction of Substituted Naphthalenes. *J. Chem. Soc. Perkin 1* **1993**, 117–120.

6 Watson, P. L.; Tulip, T. H.; Williams, I. Defluorination of Perfluoroolefins by Divalent Lanthanoid Reagents: Activating Carbon-Fluorine Bonds. *Organometallics* **1990**, *9*, 1999–2009.

7 Izod, K.; Liddle, S. T.; Clegg, W. A Convenient Route to Lanthanide Triiodide THF Solvates. Crystal Structures of $LnI_3(THF)_4$ [Ln = Pr] and $LnI_3(THF)_{3.5}$ [Ln = Nd, Gd, Y]. *Inorg. Chem.* **2004**, *43*, 214–218.

8 Janikowski, J.; Forsyth, C.; MacFarlane, D. R.; Pringle, J. M. Novel Ionic Liquids and Plastic Crystals Utilizing the Cyanate Anion. *J. Mater. Chem.* **2011**, *21*, 19219–19225.

9 Ibrahim, N.; Vilhelmsen, M. H.; Pernpointner, M.; Rominger, F.; Hashmi, A. S. K. Gold Phenolate Complexes: Synthesis, Structure, and Reactivity. *Organometallics* **2013**, *32*, 2576–2583.

10 Arashiba, K.; Kinoshita, E.; Kuriyama, S.; Eizawa, A.; Nakajima, K.; Tanaka, H.; Yoshizawa, K.; Nishibayashi, Y. Catalytic Reduction of Dinitrogen to Ammonia by Use of Molybdenum–Nitride Complexes Bearing a Tridentate Triphosphine as Catalysts. *J. Am. Chem. Soc.* **2015**, *137*, 5666–5669.

11 Ashida, Y.; Mizushima, T.; Arashiba, K.; Egi, A.; Tanaka, H.; Yoshizawa, K.; Nishibayashi, Y. Catalytic Production of Ammonia from Dinitrogen Employing Molybdenum Complexes Bearing N-Heterocyclic Carbene-based PCP-Type Pincer Ligands. Chem. Rxiv, 2022, DOI: 10.33774/chemrxiv-2002-jp6hz.

12 *CrysAlisPro*: Data Collection and Processing Software; Rigaku Corporation: Tokyo, Japan, 2015.

13 *CrystalStructure* 4.3: Crystal Structure Analysis Package, Rigaku Corporation, Tokyo, Japan, 2018.

14 Sheldrick, G. M. SHELXT – Integrated Space-Group and Crystal-Structure Determination. *Acta Crystallogr. Sect. Found. Adv.* **2015**, *71*, 3–8.

15 Sheldrick, G. M. A Short History of SHELX. *Acta Crystallogr. A* **2008**, *64*, 112–122.

16 Sheldrick, G. M. Crystal Structure Refinement with SHELXL. *Acta Crystallogr. Sect. C Struct. Chem.* **2015**, *71*, 3–8.

17 Ibers, J. A.; Hamilton, W. C. Dispersion Corrections and Crystal Structure Refinements. *Acta Crystallogr.* **1964**, *17*, 781–782.

18 Creagh, D. C.; Hubbell, J.H. *International Tables for Crystallography*; Wilson, A. J. C. Ed.; Kluwer Academic Publishers: Dordrecht, 1992; vol. C,200.

19 Creagh, D. C.; McAuley, W. J. *International Tables for Crystallography*; Wilson, A. J. C. Ed.; Kluwer

Academic Publishers: Dordrecht, 1992; vol. C, 219.

20 Maslen, E. N.; Fox, A. G.; O'Keefe, M. A. *International Tables for Crystallography*; Wilson, A. J. C. Ed.; Kluwer Academic Publishers: Dordrecht, 1992; vol. C, 500.

21 Spek, A. L. PLATON SQUEEZE: A Tool for the Calculation of the Disordered Solvent Contribution to the Calculated Structure Factors. *Acta Crystallogr. Sect. C Struct. Chem.* **2015**, *71*, 9–18.

22 Frisch, M. J.; Trucks, G. W.; Schlegel, H. B.; Scuseria, G. E.; Robb, M. A.; Cheeseman, J. R.; Scalmani, G.; Barone, V.; Petersson, G. A.; Nakatsuji, H.; Li, X.; Caricato, M.; Marenich, A. V.; Bloino, J.; Janesko, B. G.; Gomperts, R.; Mennucci, B.; Hratchian, H. P.; Ortiz, J. V.; Izmaylov, A. F.; Sonnenberg, J. L.; Williams-Young, D.; Ding, F.; Lipparini, F.; Egidi, F.; Goings, J.; Peng, B.; Petrone, A.; Henderson, T.; Ranasinghe, D.; Zakrzewski, V. G.; Gao, J.; Rega, N.; Zheng, G.; Liang, W.; Hada, M.; Ehara, M.; Toyota, K.; Fukuda, R.; Hasegawa, J.; Ishida, M.; Nakajima, T.; Honda, Y.; Kitao, O.; Nakai, H.; Vreven, T.; Throssell, K.; Montgomery, Jr., J. A.; Peralta, J. E.; Ogliaro, F.; Bearpark, M. J.; Heyd, J. J.; Brothers, E. N.; Kudin, K. N.; Staroverov, V. N.; Keith, T. A.; Kobayashi, R.; Normand, J.; Raghavachari, K.; Rendell, A. P.; Burant, J. C.; Iyengar, S. S.; Tomasi, J.; Cossi, M.; Millam, J. M.; Klene, M.; Adamo, C.; Cammi, R.; Ochterski, J. W.; Martin, R. L.; Morokuma, K.; Farkas, O.; Foresman, J. B. and Fox, D. J. Gaussian, Inc., Wallingford CT, 2019.

23 Becke, A. D. Density-functional exchange-energy approximation with correct asymptotic behavior. *Phys. Rev. A* **1988**, *38*, 3098–3100.

24 Becke, A. D. Density-functional thermochemistry. III. The role of exact exchange. *J. Chem. Phys.* **1993**, *98*, 5648–5652.S21 Spek, A. L. PLATON SQUEEZE: A Tool for the Calculation of the Disordered Solvent Contribution to the Calculated Structure Factors. *Acta Crystallogr. Sect. C Struct. Chem.* **2015**, *71*, 9–18.

25 Lee, C.; Yang, W.; Parr, R. G. Development of the Colle-Salvetti correlation-energy formula into a functional of the electron density. *Phys. Rev. B* **1988**, *37*, 785–789.

26 Vosko, S. H.; Wilk, L.; Nusair, M. J. Accurate spin-dependent electron liquid correlation energies for local spin density calculations: a critical analysis. *Can. J. Phys.* **1980**, *58*, 1200–1211.

27 Grimme, S.; Antony, J.; Ehrlich, S.; Krieg, H. A consistent and accurate ab initio parametrization of density functional dispersion correction (DFT-D) for the 94 elements H-Pu. *J. Phys. Chem.* **2010**, *132*, 154104.

28 Dolg, M.; Wedig, U.; Stoll, H.; Preuß, H. Energy-adjusted ab initio pseudopotentials for the first row transition elements. *J. Chem. Phys*. **1987**, *86*, 866–872.

29 Andrae, D.; Häußermann, U.; Dolg, M.; Stoll, H.; Preuß, H. Energy-adjustedab initio pseudopotentials for the second and third row transition elements. *Theor. Chim. Acta.* **1990**, *77*, 123–141.

30 Ditchfield, R.; Hehre, W. J.; Pople, J. A. Self-consistent molecular-orbital methods. IX. An extended Gaussian-type basis for molecular-orbital studies of organic molecules. *J. Chem. Phys*. **1971**, *54*, 724–728.

31 Hehre, W. J.; Ditchfield, R.; Pople, J. A. Self—consistent molecular orbital methods. XII. Further extensions of Gaussian—type basis sets for use in molecular orbital studies of organic molecules. *J. Chem. Phys.* **1972**, *56*, 2257–2261.

32 Hariharan, P. C.; Pople, J. A. The influence of polarization functions on molecular orbital hydrogenation energies. *Theor. Chem. Acc.* **1973**, *28*, 213–222.

33 Francl, M. M.; Pietro, W. J.; Hehre, W. J.; Binkley, J. S.; Gordon, M. S.; DeFrees, D. J.; Pople, J. A. Self-consistent molecular orbital methods. XXIII. A polarization-type basis set for second-row elements. *J. Chem. Phys*. **1982**, *77*, 3654–3665.

34 Fukui, K. Formulation of the reaction coordinate. *J. Phys. Chem.* **1970**, *74*, 4161–4163.

35 Fukui, K. The path of chemical reactions - the IRC approach. *Acc. Chem. Res.* **1981**, *14*, 363–368.

36 Gonzalez, C.; Schlegel, H. B. Reaction path following in mass-weighted internal coordinates. *J. Phys. Chem.* **1990**, *94*, 5523–5527.

37 Krishnan, R.; Binkley, J. S.; Seeger, R.; Pople, J. A. Self-consistent molecular orbital methods. XX. A basis set for correlated wave functions. *J. Chem. Phys.* **1980**, *72*, 650–654.

38 McLean, A. D.; Chandler, G. S. Contracted Gaussian basis sets for molecular calculations. I. Second row atoms, Z=11–18. *J. Chem. Phys.* **1980**, *72*, 5639–5648.

39 Clark, T.; Chandrasekhar, J.; Spitznagel, G. W.; Schleyer, P. v. R. Efficient diffuse function-augmented basis sets for anion calculations. III. The 3-21+G basis set for first-row elements, Li–F. *J. Comput. Chem.* **1983**, *4*, 294–301.

40 Tomasi, J.; Mennucci, B.; Cammi, R. Quantum mechanical continuum solvation models. *Chem. Rev*. **2005**, *105*, 2999–3094.

41 Evans, W. J.; Gummersheimer, T. S.; Ziller J. W. Coordination Chemistry of Samarium Diiodide with Ethers Including the Crystal Structure of Tetrahydrofuran-Solvated Samarium Diiodide, $SmI_2(THF)_5$. *J. Am. Chem. Soc.* **1995**, *117*, 8999–9002.
