# Supplementary material for: Direct synthesis of cyanate anion from dinitrogen catalysed by molybdenum complexes bearing pincer-type ligand
Source: Nat Commun. 2022 Oct 24;13:6161. doi: 10.1038/s41467-022-33809-5 (PMC9592615; doi:10.1038/s41467-022-33809-5)
Supplement: Supplementary file 3 — Description of Additional Supplementary Files [file 41467_2022_33809_MOESM3_ESM.pdf]

Supplementary Data 1: Information of Cartesian Coordinates
